# Supplementary material for: Identification of a conserved virion-stabilizing network inside the interprotomer pocket of enteroviruses
Source: Commun Biol. 2021 Feb 26;4:250. doi: 10.1038/s42003-021-01779-x (PMC7910612; doi:10.1038/s42003-021-01779-x)
Supplement: Supplementary file 2 — Supplementary Information [file 42003_2021_1779_MOESM2_ESM.pdf]

Supplementary Information

Identification of a conserved virion-stabilizing network inside the interprotomer pocket of enteroviruses

Justin W. Flatt, Aušra Domanska, Alma L. Seppälä, Sarah J. Butcher*

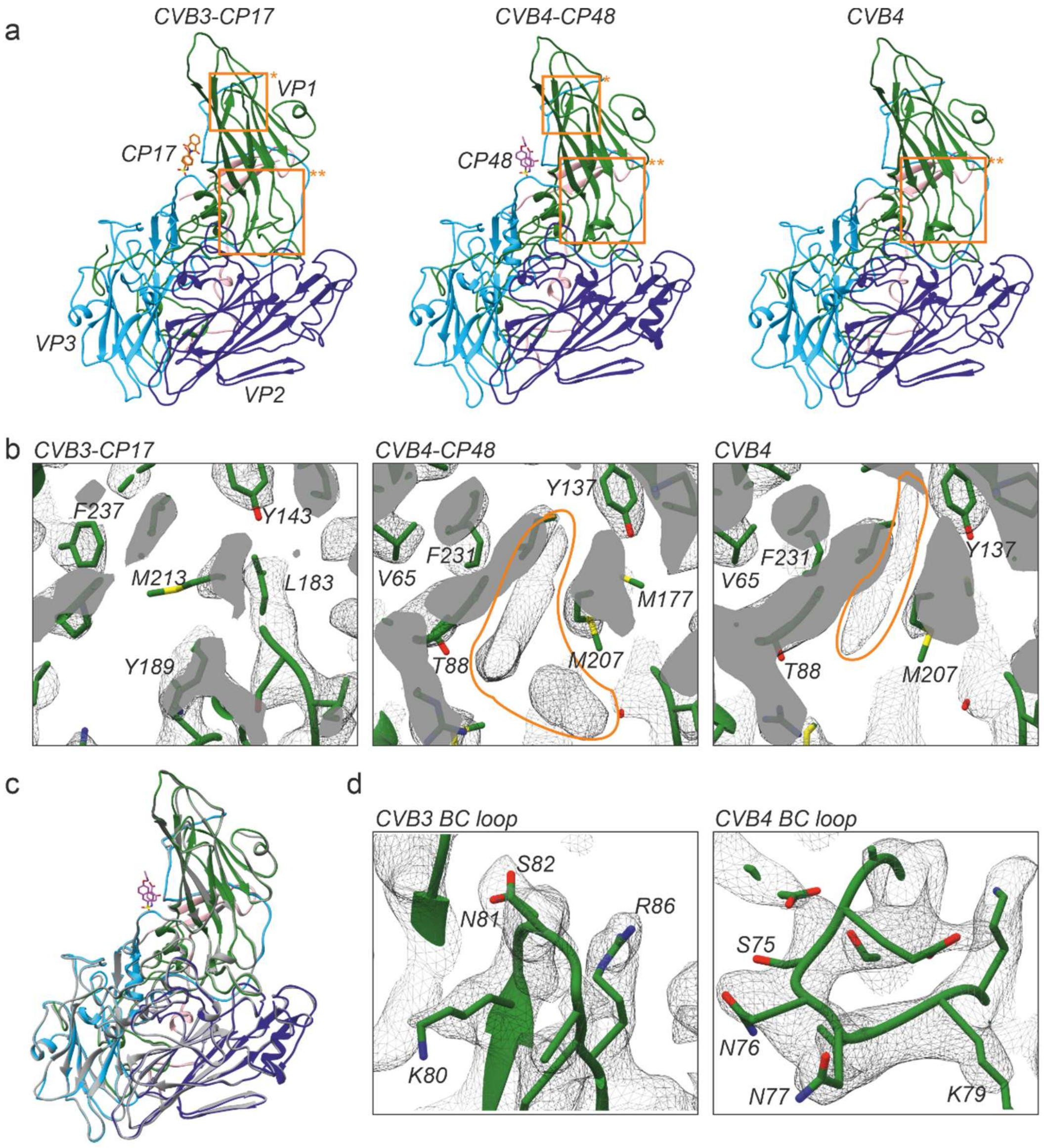

Supplementary Figure 1. Comparison between CVB3 and CVB4 structures. (a) Atomic models of CVB3-CP17, CVB4-CP48 and CVB4 alone. Boxed areas denoted with two asterisks are zoomed in (b) and with one asterisk in (d). (b) Density at the hydrophobic pocket of VP1 in CVB3-CP17, CVB4-CP48 and CVB4 alone. Notably, the hydrophobic pocket in CVB3 Nancy strain used in the study is empty. Density attributed to lipid factor in CP48-CVB4 and CVB4 reconstructions is encircled in orange.(c) Overlay of atomic models of CP48-CVB4 complex

(VP1, green; VP2, blue; VP3, light blue; VP4 light pink; CP48, magenta) with a control structure of CVB4 alone (gray); RMSD between the two structures is 0.45 Å. (d) Zoomed in view of BC loops in CVB3-CP17 and CVB4-CP48 reconstructions.

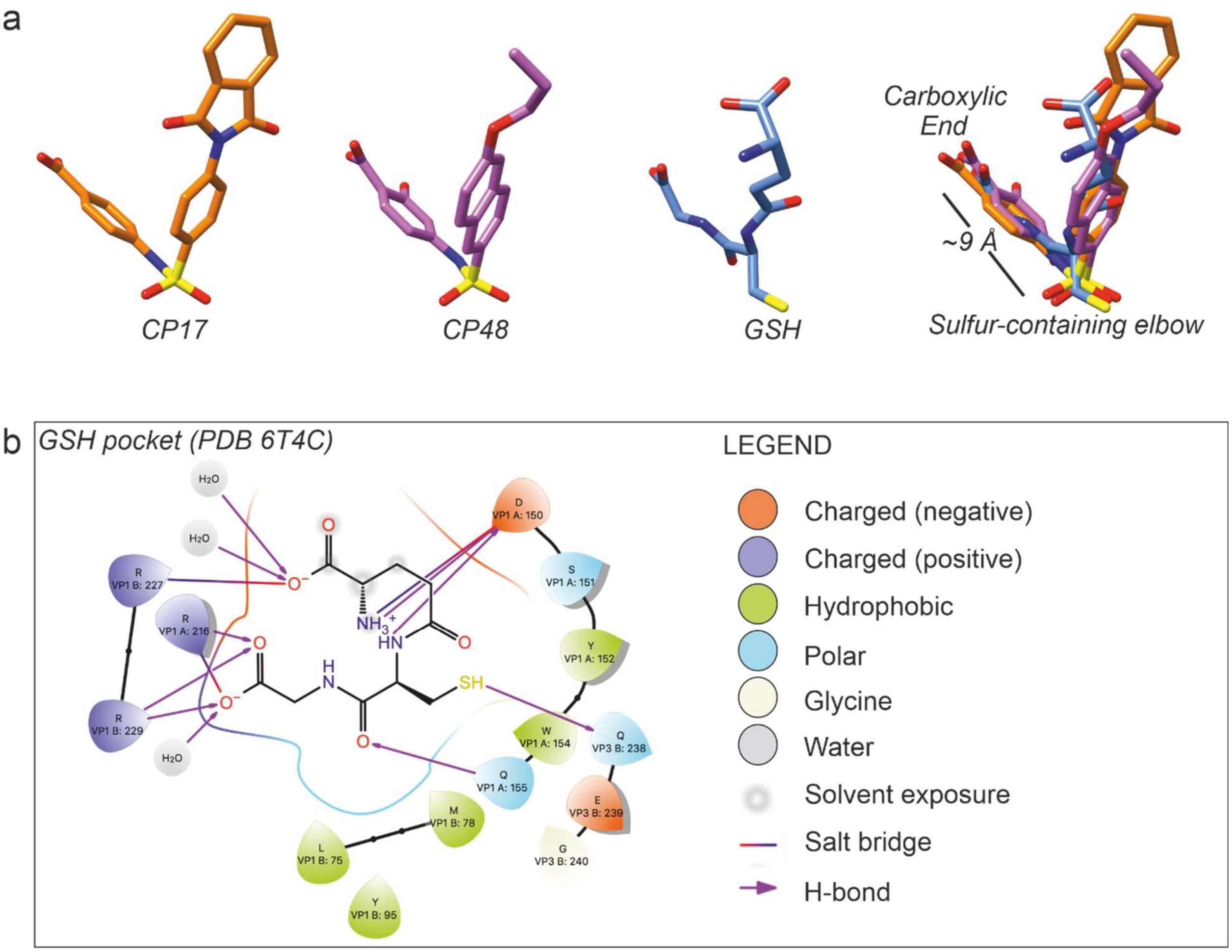

Supplementary Figure 2. Glutathione (GSH) in comparison to CP17 and CP48. (a) Interprotomer-targeting molecules have similar features. (b) Interaction diagram of GSH with EVF3 viral proteins (PDB 6T4C) generated in Schrödinger Maestro software v12.02.

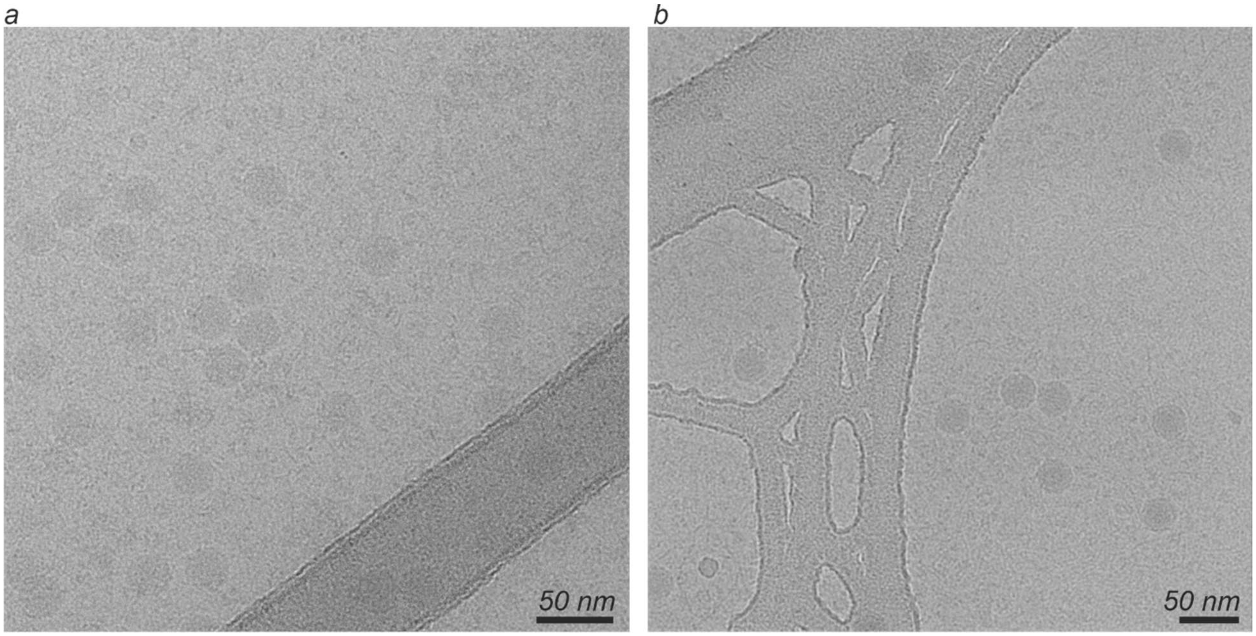

Supplementary Figure 3. Representative aligned micrographs of (a) CVB4-CP48 and (b) CVB4. Scale bar, 50 nm.

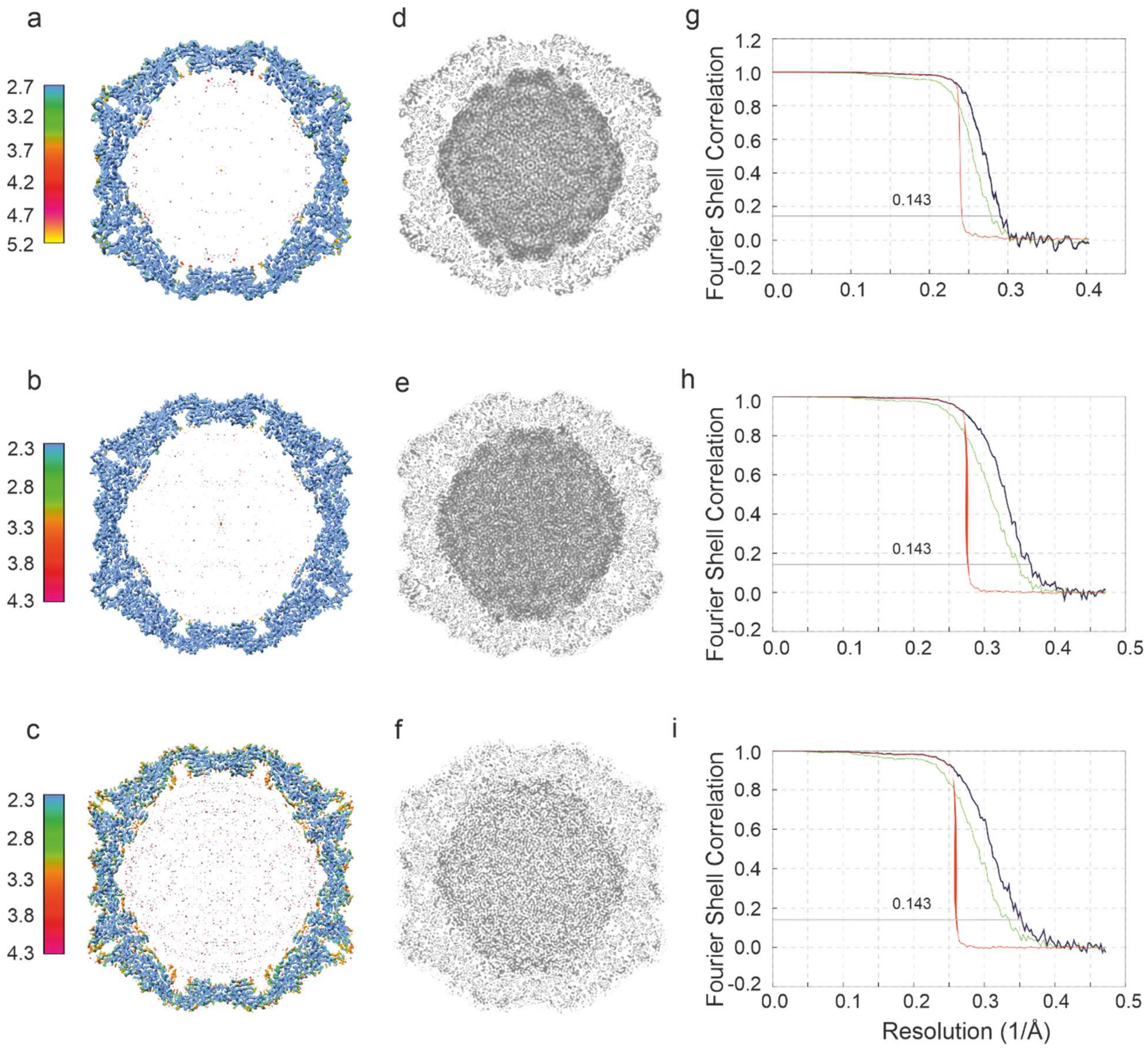

Supplementary Figure 4: (a-c) ResMap resolution slices with colour key showing resolution in Å, (d-f) central cross-sections of the reconstructions (g-i) FSC plots where the black curve is the plot for the corrected FSC, blue is for masked maps, red is for phase randomized masked maps and green is for unmasked maps, output from RELION 3. (a, d, g) CVB4, (b, e, h) CVB4-CP48, and (c,f,i) CVB3-CP17.

WORLDWIDE PDB PROTEIN DATA BANK

Full wwPDB EM Map/Model Validation Report (i)

Jul 3, 2020 – 11:20 AM BST

PDB ID	:	6ZCL
EMDB ID	:	EMD-11166
Title	:	Coxsackievirus B3 in complex with capsid binder compound 17
Deposited on	:	2020-06-11
Resolution	:	2.80 Å(reported)

This is a Full wwPDB EM Map/Model Validation Report.

This report is produced by the wwPDB biocuration pipeline after annotation of the structure.

We welcome your comments at *validation@mail.wwpdb.org*
A user guide is available at
https://www.wwpdb.org/validation/2017/EMValidationReportHelp
with specific help available everywhere you see the (i) symbol.

CONFIDENTIAL VALIDATION REPORT

The following versions of software and data (see references (i)) were used in the production of this report:

EMDB validation analysis	:	0.0.0.dev33
Mogul	:	1.8.5 (274361), CSD as541be (2020)
MolProbity	:	4.02b-467
buster-report	:	1.1.7 (2018)
Percentile statistics	:	20191225.v01 (using entries in the PDB archive December 25th 2019)
Ideal geometry (proteins)	:	Engh & Huber (2001)
Ideal geometry (DNA, RNA)	:	Parkinson et al. (1996)
Validation Pipeline (wwPDB-VP)	:	2.13.dev1

1 Overall quality at a glance (i)

The following experimental techniques were used to determine the structure:
ELECTRON MICROSCOPY

The reported resolution of this entry is 2.80 Å.

Percentile scores (ranging between 0-100) for global validation metrics of the entry are shown in the following graphic. The table shows the number of entries on which the scores are based.

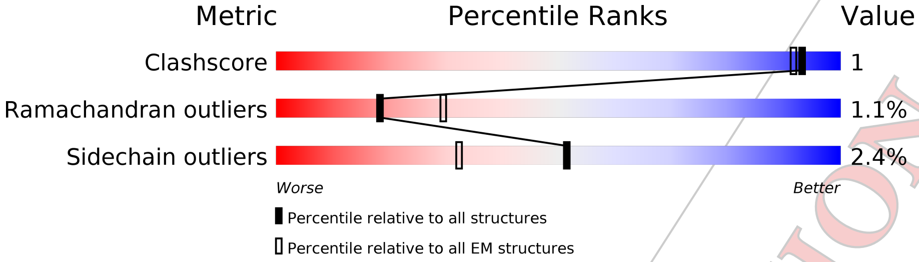

Metric	Whole archive (#Entries)	EM structures (#Entries)
Clashscore	158937	4297
Ramachandran outliers	154571	4023
Sidechain outliers	154315	3826

The table below summarises the geometric issues observed across the polymeric chains and their fit to the map. The red, orange, yellow and green segments on the bar indicate the fraction of residues that contain outliers for >=3, 2, 1 and 0 types of geometric quality criteria respectively. A grey segment represents the fraction of residues that are not modelled. The numeric value for each fraction is indicated below the corresponding segment, with a dot representing fractions <=5% The upper red bar (where present) indicates the fraction of residues that have poor fit to the EM map (all atom inclusion < 40%). The numeric value is given above the bar.

Mol	Chain	Length	Quality of chain
1	A	269	• 96% •
2	B	252	• 94% 6%
3	C	237	• 97% •
4	D	68	9% 76% • 19%

The following table lists non-polymeric compounds, carbohydrate monomers and non-standard residues in protein, DNA, RNA chains that are outliers for geometric or electron-density-fit criteria:

Mol	Type	Chain	Res	Chirality	Geometry	Clashes	Electron density
6	MYR	D	101	-	-	X	-

CONFIDENTIAL VALIDATION REPORT

2 Entry composition (i)

There are 6 unique types of molecules in this entry. The entry contains 6370 atoms, of which 0 are hydrogens and 0 are deuteriums.

In the tables below, the AltConf column contains the number of residues with at least one atom in alternate conformation and the Trace column contains the number of residues modelled with at most 2 atoms.

- Molecule 1 is a protein called capsid protein VP1.

Mol	Chain	Residues	Atoms					AltConf	Trace
			Total	C	N	O	S		
1	A	269	2133	1346	376	403	8	0	0

- Molecule 2 is a protein called capsid protein VP2.

Mol	Chain	Residues	Atoms					AltConf	Trace
			Total	C	N	O	S		
2	B	252	1935	1222	327	370	16	0	0

- Molecule 3 is a protein called capsid protein VP3.

Mol	Chain	Residues	Atoms					AltConf	Trace
			Total	C	N	O	S		
3	C	237	1828	1170	293	348	17	0	0

- Molecule 4 is a protein called capsid protein VP4.

Mol	Chain	Residues	Atoms					AltConf	Trace
			Total	C	N	O	S		
4	D	55	429	267	74	87	1	0	0

- Molecule 5 is 4-[[4-[1,3-bis(oxidanylidene)isoindol-2-yl]phenyl]sulfonylamino]benzoic acid (three-letter code: FHK) (formula: $C_{21}H_{14}N_2O_6S$) (labeled as "Ligand of Interest" by author).

CONFIDENTIAL VALIDATION REPORT

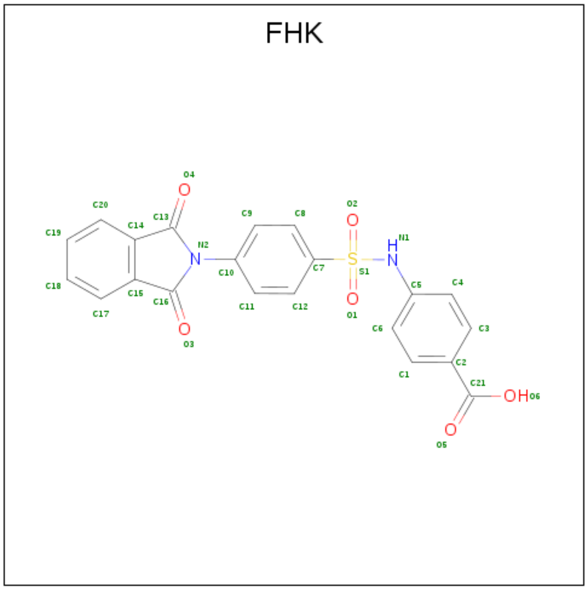

Mol	Chain	Residues	Atoms					AltConf
			Total	C	N	O	S	
5	C	1	30	21	2	6	1	0

- Molecule 6 is MYRISTIC ACID (three-letter code: MYR) (formula: $C_{14}H_{28}O_2$).

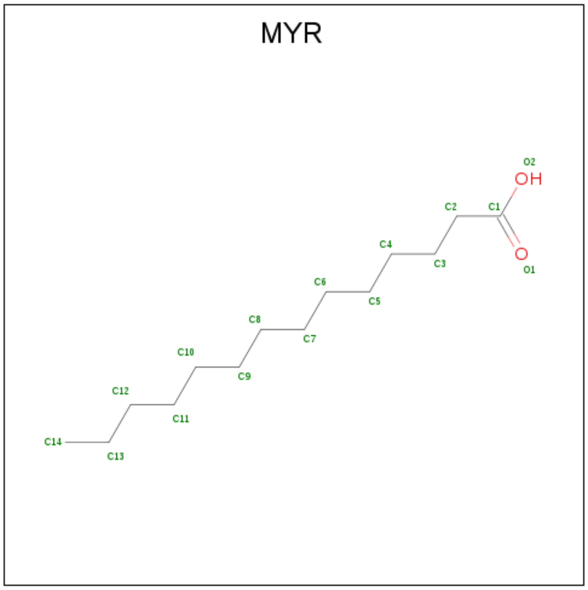

Mol	Chain	Residues	Atoms			AltConf
			Total	C	O	
6	D	1	15	14	1	0

CONFIDENTIAL VALIDATION REPORT

3 Residue-property plots ⓘ

These plots are drawn for all protein, RNA, DNA and oligosaccharide chains in the entry. The first graphic for a chain summarises the proportions of the various outlier classes displayed in the second graphic. The second graphic shows the sequence view annotated by issues in geometry and atom inclusion in map density. Residues are color-coded according to the number of geometric quality criteria for which they contain at least one outlier: green = 0, yellow = 1, orange = 2 and red = 3 or more. A red diamond above a residue indicates a poor fit to the EM map for this residue (all atom inclusion < 40%). Stretches of 2 or more consecutive residues without any outlier are shown as a green connector. Residues present in the sample, but not in the model, are shown in grey.

- Molecule 1: capsid protein VP1

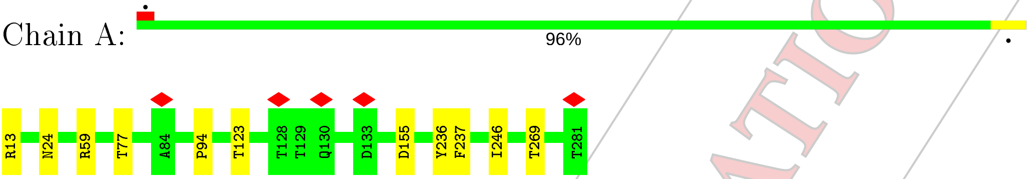

- Molecule 2: capsid protein VP2

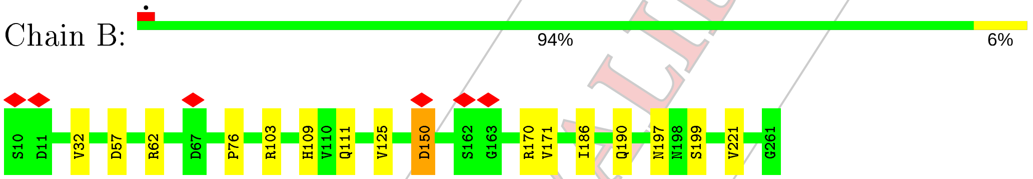

- Molecule 3: capsid protein VP3

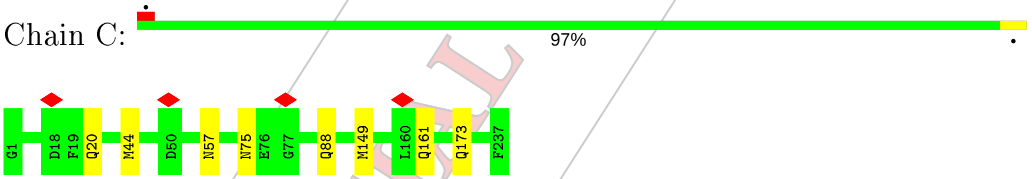

- Molecule 4: capsid protein VP4

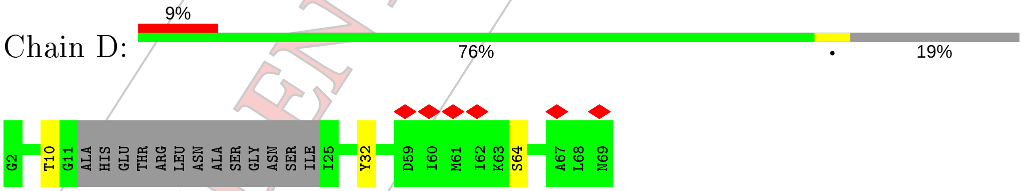

CONFIDENTIAL VALIDATION REPORT

4 Experimental information (i)

Property	Value	Source
EM reconstruction method	SINGLE PARTICLE	Depositor
Imposed symmetry	POINT, Not provided	Depositor
Number of particles used	18626	Depositor
Resolution determination method	FSC 0.143 CUT-OFF	Depositor
CTF correction method	PHASE FLIPPING AND AMPLITUDE CORRECTION	Depositor
Microscope	FEI TITAN KRIOS	Depositor
Voltage (kV)	300	Depositor
Electron dose ($e^-/\text{Å}^2$)	47	Depositor
Minimum defocus (nm)	Not provided	Depositor
Maximum defocus (nm)	Not provided	Depositor
Magnification	Not provided	Depositor
Image detector	GATAN K2 SUMMIT (4k x 4k)	Depositor
Maximum map value	0.125	Depositor
Minimum map value	-0.070	Depositor
Average map value	0.001	Depositor
Map value standard deviation	0.007	Depositor
Recommended contour level	0.019	Depositor
Map size (Å)	445.19998, 445.19998, 445.19998	Depositor
Map dimensions	420, 420, 420	Depositor
Map angles (°)	90.0, 90.0, 90.0	Depositor
Pixel spacing (Å)	1.06, 1.06, 1.06	Depositor

CONFIDENTIAL VALIDATION REPORT

5 Model quality (i)

5.1 Standard geometry (i)

Bond lengths and bond angles in the following residue types are not validated in this section: MYR, FHK

The Z score for a bond length (or angle) is the number of standard deviations the observed value is removed from the expected value. A bond length (or angle) with $|Z| > 5$ is considered an outlier worth inspection. RMSZ is the root-mean-square of all Z scores of the bond lengths (or angles).

Mol	Chain	Bond lengths		Bond angles	
		RMSZ	#|Z| >5	RMSZ	#|Z| >5
1	A	0.87	0/2190	1.02	2/2986 (0.1%)
2	B	0.85	0/1985	1.04	1/2716 (0.0%)
3	C	0.86	0/1879	0.99	1/2560 (0.0%)
4	D	0.86	0/436	0.98	0/587
All	All	0.86	0/6490	1.01	4/8849 (0.0%)

Chiral center outliers are detected by calculating the chiral volume of a chiral center and verifying if the center is modelled as a planar moiety or with the opposite hand.A planarity outlier is detected by checking planarity of atoms in a peptide group, atoms in a mainchain group or atoms of a sidechain that are expected to be planar.

Mol	Chain	#Chirality outliers	#Planarity outliers
1	A	0	2
2	B	0	1
All	All	0	3

There are no bond length outliers.

All (4) bond angle outliers are listed below:

Mol	Chain	Res	Type	Atoms	Z	Observed(°)	Ideal(°)
3	C	161	GLN	N-CA-CB	5.42	120.35	110.60
1	A	237	PHE	CB-CG-CD1	5.30	124.51	120.80
2	B	150	ASP	N-CA-CB	5.26	120.06	110.60
1	A	237	PHE	CB-CG-CD2	-5.21	117.15	120.80

There are no chirality outliers.

All (3) planarity outliers are listed below:

CONFIDENTIAL VALIDATION REPORT

Mol	Chain	Res	Type	Group
1	A	236	TYR	Sidechain
1	A	59	ARG	Sidechain
2	B	103	ARG	Sidechain

5.2 Too-close contacts (i)

In the following table, the Non-H and H(model) columns list the number of non-hydrogen atoms and hydrogen atoms in the chain respectively. The H(added) column lists the number of hydrogen atoms added and optimized by MolProbity. The Clashes column lists the number of clashes within the asymmetric unit, whereas Symm-Clashes lists symmetry related clashes.

Mol	Chain	Non-H	H(model)	H(added)	Clashes	Symm-Clashes
1	A	2133	0	2070	3	0
2	B	1935	0	1871	2	0
3	C	1828	0	1762	1	0
4	D	429	0	415	11	0
5	C	30	0	0	0	0
6	D	15	0	27	10	0
All	All	6370	0	6145	16	0

The all-atom clashscore is defined as the number of clashes found per 1000 atoms (including hydrogen atoms). The all-atom clashscore for this structure is 1.

All (16) close contacts within the same asymmetric unit are listed below, sorted by their clash magnitude.

Atom-1	Atom-2	Interatomic distance (Å)	Clash overlap (Å)
4:D:32:TYR:HD1	6:D:101:MYR:C13	1.34	1.39
4:D:32:TYR:CD1	6:D:101:MYR:C13	2.19	1.24
4:D:32:TYR:CD1	6:D:101:MYR:H131	1.75	1.21
4:D:32:TYR:CD1	6:D:101:MYR:H132	1.83	1.14
4:D:32:TYR:HD1	6:D:101:MYR:H131	0.90	1.04
1:A:269:THR:CA	1:A:269:THR:CG2	2.51	0.88
1:A:269:THR:CG2	1:A:269:THR:OG1	2.42	0.67
1:A:269:THR:CA	1:A:269:THR:OG1	2.46	0.64
4:D:32:TYR:HE1	6:D:101:MYR:C11	2.16	0.58
4:D:32:TYR:HE1	6:D:101:MYR:H112	1.74	0.51
4:D:32:TYR:CE1	6:D:101:MYR:H112	2.48	0.49
4:D:32:TYR:CE1	6:D:101:MYR:C11	2.96	0.49
4:D:32:TYR:CE1	6:D:101:MYR:C13	2.90	0.47
3:C:20:GLN:HE22	4:D:32:TYR:H	1.64	0.44
2:B:109:HIS:CE1	2:B:199:SER:HB3	2.57	0.40

Continued on next page...

CONFIDENTIAL VALIDATION REPORT

Continued from previous page...

Atom-1	Atom-2	Interatomic distance (Å)	Clash overlap (Å)
2:B:32:VAL:HG23	2:B:190:GLN:HE21	1.86	0.40

There are no symmetry-related clashes.

5.3 Torsion angles ⓘ

5.3.1 Protein backbone ⓘ

In the following table, the Percentiles column shows the percent Ramachandran outliers of the chain as a percentile score with respect to all PDB entries followed by that with respect to all EM entries.

The Analysed column shows the number of residues for which the backbone conformation was analysed, and the total number of residues.

Mol	Chain	Analysed	Favoured	Allowed	Outliers	Percentiles	
1	A	267/269 (99%)	251 (94%)	13 (5%)	3 (1%)	14	41
2	B	250/252 (99%)	234 (94%)	13 (5%)	3 (1%)	13	39
3	C	235/237 (99%)	221 (94%)	12 (5%)	2 (1%)	17	46
4	D	51/68 (75%)	48 (94%)	2 (4%)	1 (2%)	7	24
All	All	803/826 (97%)	754 (94%)	40 (5%)	9 (1%)	18	41

All (9) Ramachandran outliers are listed below:

Mol	Chain	Res	Type
2	B	150	ASP
3	C	57	ASN
1	A	155	ASP
2	B	57	ASP
2	B	76	PRO
1	A	246	ILE
4	D	64	SER
1	A	94	PRO
3	C	88	GLN

5.3.2 Protein sidechains ⓘ

In the following table, the Percentiles column shows the percent sidechain outliers of the chain as a percentile score with respect to all PDB entries followed by that with respect to all EM

CONFIDENTIAL VALIDATION REPORT

entries.

The Analysed column shows the number of residues for which the sidechain conformation was analysed, and the total number of residues.

Mol	Chain	Analysed	Rotameric	Outliers	Percentiles	
1	A	235/236 (100%)	231 (98%)	4 (2%)	60	87
2	B	214/214 (100%)	206 (96%)	8 (4%)	34	68
3	C	205/205 (100%)	201 (98%)	4 (2%)	55	84
4	D	47/57 (82%)	46 (98%)	1 (2%)	53	84
All	All	701/712 (98%)	684 (98%)	17 (2%)	51	81

All (17) residues with a non-rotameric sidechain are listed below:

Mol	Chain	Res	Type
1	A	13	ARG
1	A	24	ASN
1	A	77	THR
1	A	123	THR
2	B	62	ARG
2	B	111	GLN
2	B	125	VAL
2	B	170	ARG
2	B	171	VAL
2	B	186	ILE
2	B	197	ASN
2	B	221	VAL
3	C	44	MET
3	C	75	ASN
3	C	149	MET
3	C	173	GLN
4	D	10	THR

Some sidechains can be flipped to improve hydrogen bonding and reduce clashes. All (9) such sidechains are listed below:

Mol	Chain	Res	Type
1	A	24	ASN
1	A	41	GLN
1	A	164	ASN
1	A	211	ASN
1	A	220	HIS
2	B	70	GLN

Continued on next page...

CONFIDENTIAL VALIDATION REPORT

Continued from previous page...

Mol	Chain	Res	Type
2	B	109	HIS
2	B	197	ASN
3	C	20	GLN

5.3.3 RNA (i)

There are no RNA molecules in this entry.

5.4 Non-standard residues in protein, DNA, RNA chains (i)

There are no non-standard protein/DNA/RNA residues in this entry.

5.5 Carbohydrates (i)

There are no carbohydrates in this entry.

5.6 Ligand geometry (i)

2 ligands are modelled in this entry.

In the following table, the Counts columns list the number of bonds (or angles) for which Mogul statistics could be retrieved, the number of bonds (or angles) that are observed in the model and the number of bonds (or angles) that are defined in the Chemical Component Dictionary. The Link column lists molecule types, if any, to which the group is linked. The Z score for a bond length (or angle) is the number of standard deviations the observed value is removed from the expected value. A bond length (or angle) with $|Z| > 2$ is considered an outlier worth inspection. RMSZ is the root-mean-square of all Z scores of the bond lengths (or angles).

Mol	Type	Chain	Res	Link	Bond lengths			Bond angles		
					Counts	RMSZ	$\#\|Z\| > 2$	Counts	RMSZ	$\#\|Z\| > 2$
5	FHK	C	301	-	31,33,33	1.45	3 (9%)	46,49,49	1.66	7 (15%)
6	MYR	D	101	4	14,14,15	0.28	0	13,13,15	1.14	2 (15%)

In the following table, the Chirals column lists the number of chiral outliers, the number of chiral centers analysed, the number of these observed in the model and the number defined in the Chemical Component Dictionary. Similar counts are reported in the Torsion and Rings columns. '-' means no outliers of that kind were identified.

Mol	Type	Chain	Res	Link	Chirals	Torsions	Rings
5	FHK	C	301	-	-	5/15/35/35	0/4/4/4

Continued on next page...

CONFIDENTIAL VALIDATION REPORT

Continued from previous page...

Mol	Type	Chain	Res	Link	Chirals	Torsions	Rings
6	MYR	D	101	4	-	3/11/12/13	-

All (3) bond length outliers are listed below:

Mol	Chain	Res	Type	Atoms	Z	Observed(Å)	Ideal(Å)
5	C	301	FHK	S1-N1	5.88	1.73	1.63
5	C	301	FHK	C14-C13	-2.23	1.45	1.48
5	C	301	FHK	C15-C16	-2.12	1.45	1.48

All (9) bond angle outliers are listed below:

Mol	Chain	Res	Type	Atoms	Z	Observed(^{o})	Ideal(^{o})
5	C	301	FHK	O3-C16-N2	4.62	131.60	125.19
5	C	301	FHK	O4-C13-N2	4.61	131.58	125.19
5	C	301	FHK	O3-C16-C15	-3.66	121.56	128.68
5	C	301	FHK	O4-C13-C14	-3.50	121.87	128.68
5	C	301	FHK	C7-S1-N1	-3.45	102.50	106.83
5	C	301	FHK	C3-C2-C21	-3.18	116.09	120.37
6	D	101	MYR	C7-C6-C5	-2.61	101.17	114.42
5	C	301	FHK	C1-C2-C21	2.36	123.54	120.37
6	D	101	MYR	C12-C11-C10	-2.28	102.85	114.42

There are no chirality outliers.

All (8) torsion outliers are listed below:

Mol	Chain	Res	Type	Atoms
6	D	101	MYR	C1-C2-C3-C4
5	C	301	FHK	C9-C10-N2-C13
5	C	301	FHK	C11-C10-N2-C13
5	C	301	FHK	C9-C10-N2-C16
5	C	301	FHK	C11-C10-N2-C16
6	D	101	MYR	C7-C8-C9-C10
6	D	101	MYR	C6-C7-C8-C9
5	C	301	FHK	C12-C7-S1-O2

There are no ring outliers.

1 monomer is involved in 10 short contacts:

Mol	Chain	Res	Type	Clashes	Symm-Clashes
6	D	101	MYR	10	0

CONFIDENTIAL VALIDATION REPORT

The following is a two-dimensional graphical depiction of Mogul quality analysis of bond lengths, bond angles, torsion angles, and ring geometry for all instances of the Ligand of Interest. In addition, ligands with molecular weight > 250 and outliers as shown on the validation Tables will also be included. For torsion angles, if less then 5% of the Mogul distribution of torsion angles is within 10 degrees of the torsion angle in question, then that torsion angle is considered an outlier. Any bond that is central to one or more torsion angles identified as an outlier by Mogul will be highlighted in the graph. For rings, the root-mean-square deviation (RMSD) between the ring in question and similar rings identified by Mogul is calculated over all ring torsion angles. If the average RMSD is greater than 60 degrees and the minimal RMSD between the ring in question and any Mogul-identified rings is also greater than 60 degrees, then that ring is considered an outlier. The outliers are highlighted in purple. The color gray indicates Mogul did not find sufficient equivalents in the CSD to analyse the geometry.

Ligand FHK C 301

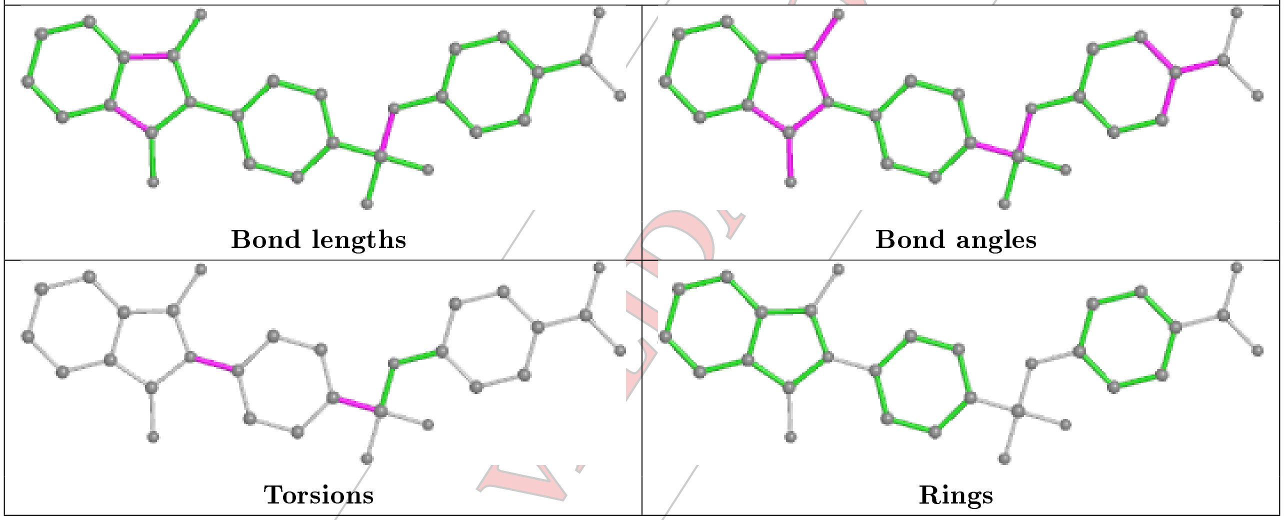

5.7 Other polymers (i)

There are no such residues in this entry.

5.8 Polymer linkage issues (i)

There are no chain breaks in this entry.

6 Map visualisation (i)

This section contains visualisations of the EMDB entry EMD-11166. These are intended to permit visual inspection of the internal detail of the map and identification of artifacts.

6.1 Orthogonal projections (i)

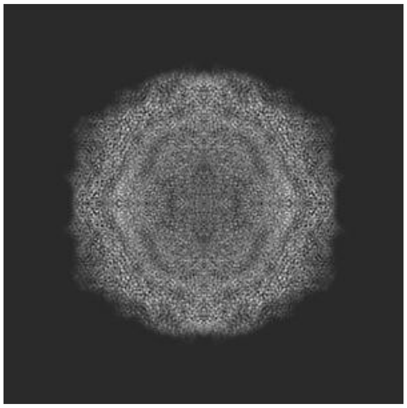

X

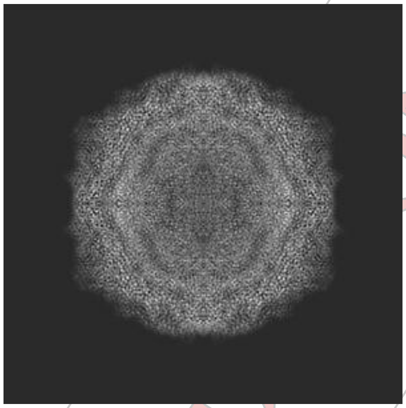

Y

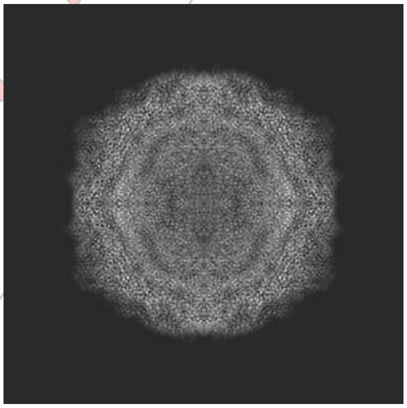

Z

The images above show the map projected in three orthogonal projections, in greyscale.

6.2 Central slices (i)

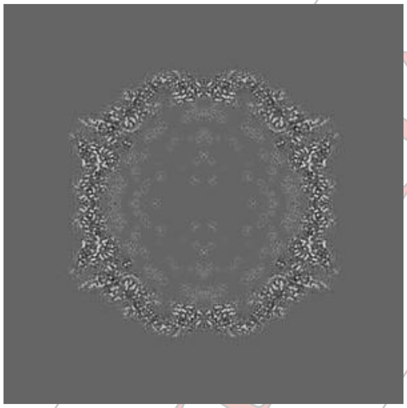

X

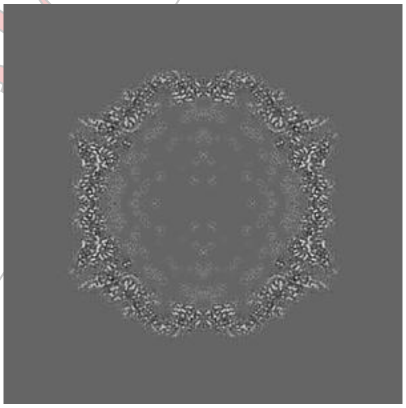

Y

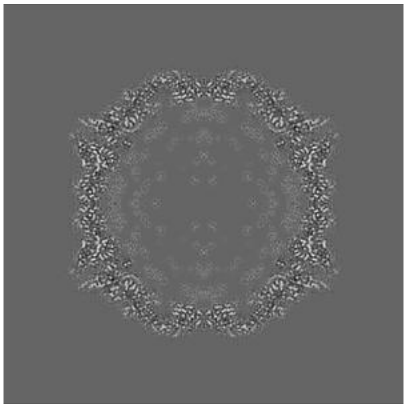

Z

The images above show central slices of the map in three orthogonal directions, in greyscale.

CONFIDENTIAL VALIDATION REPORT

6.3 Largest variance slices (i)

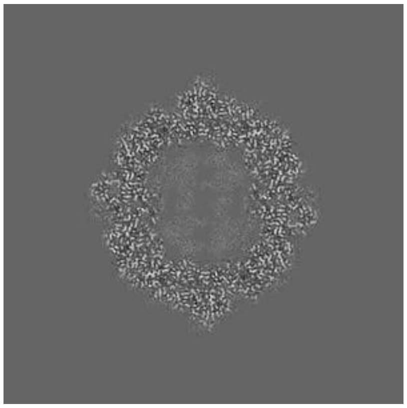

X Index: 125

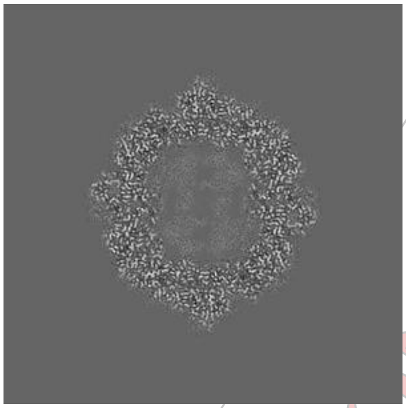

Y Index: 125

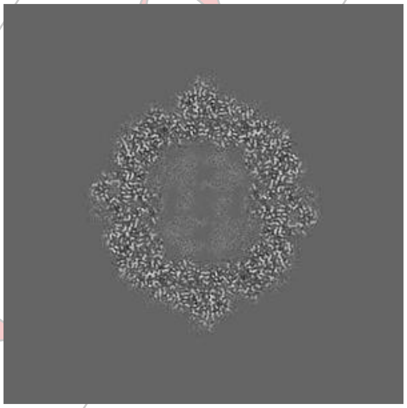

Z Index: 125

The images above show the highest variance slices of the map in three orthogonal directions, in greyscale.

6.4 Orthogonal surface views (i)

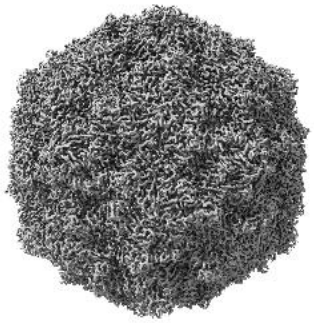

X

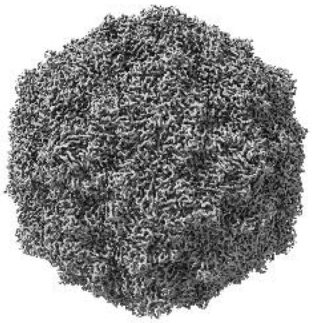

Y

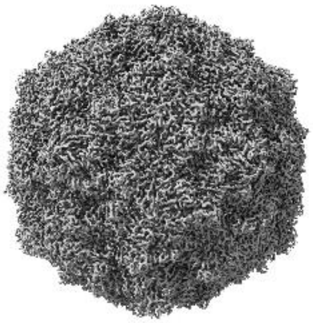

Z

The images above show the 3D surface view of the map at the recommended contour level 0.019. This in conjunction with the slice images can indicate whether an appropriate contour level has been selected.

6.5 Mask visualisation (i)

This section was not generated. No masks were provided.

CONFIDENTIAL VALIDATION REPORT

7 Map analysis (i)

This section contains the results of statistical analysis of the map.

7.1 Map-value distribution (i)

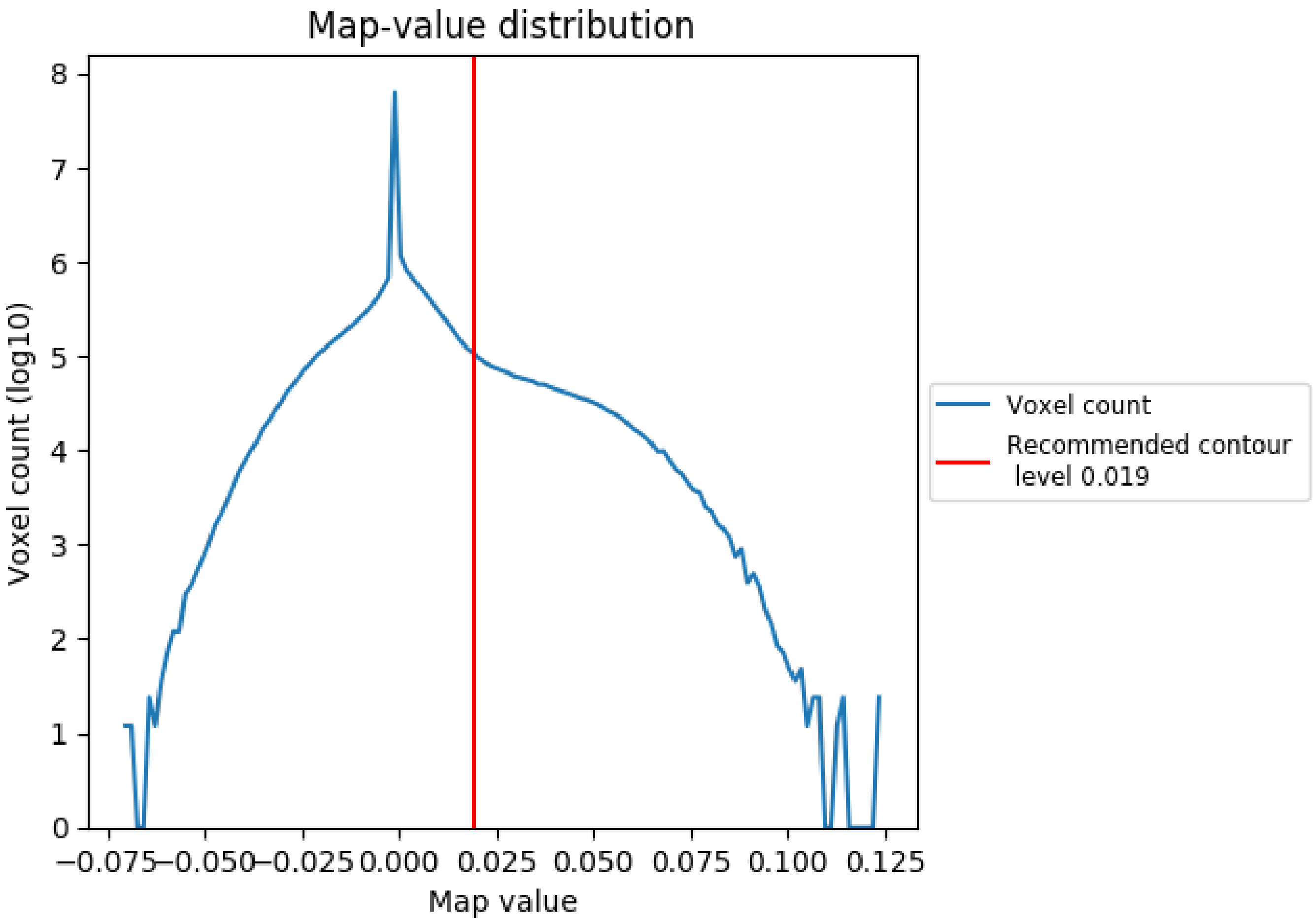

The map-value distribution is plotted in 128 intervals along the x-axis. The y-axis is logarithmic. A spike in this graph at zero usually indicates that the volume has been masked.

CONFIDENTIAL REPORT

7.2 Volume estimate ⓘ

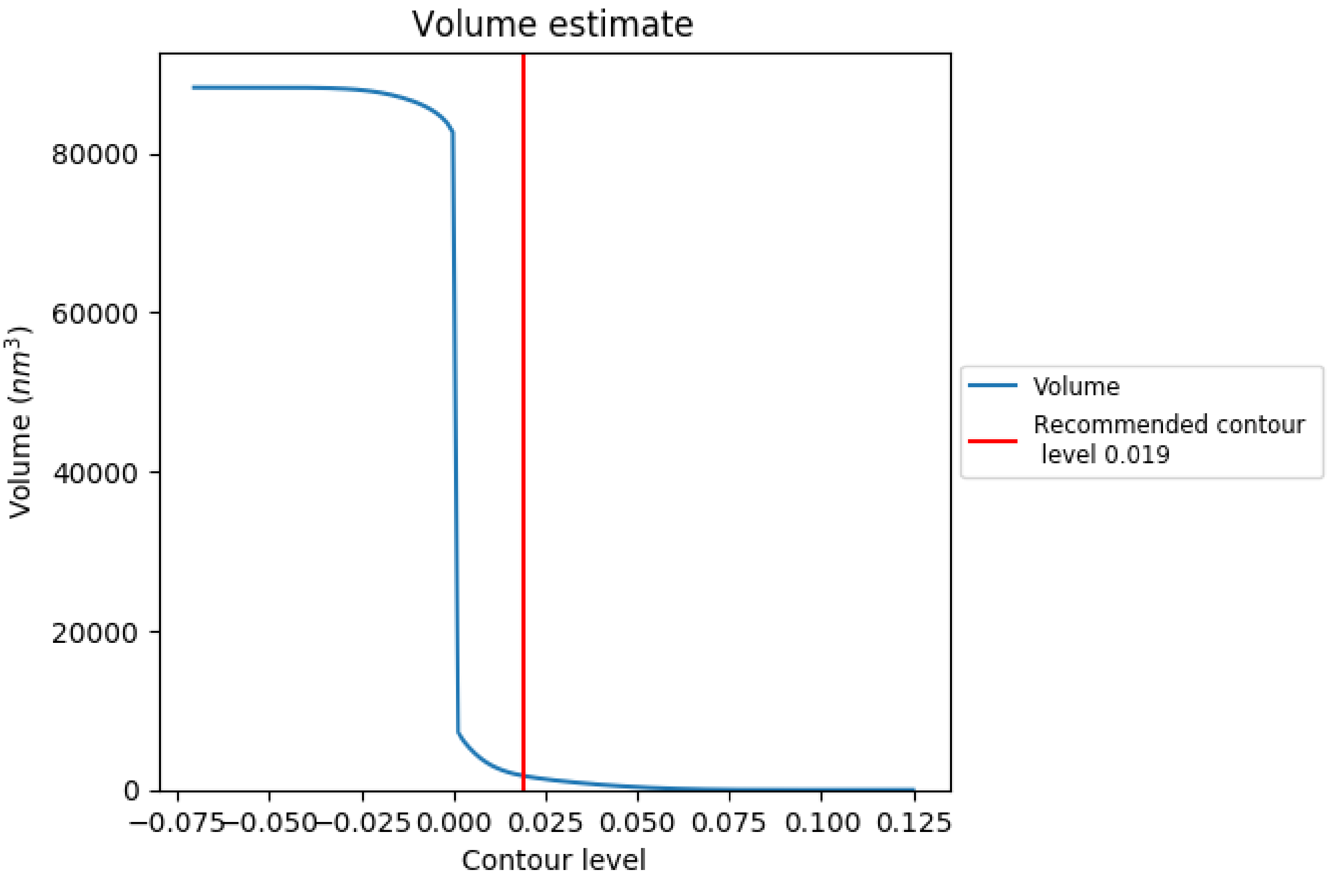

The volume at the recommended contour level is 1776 nm^3; this corresponds to an approximate mass of 1604 kDa.

The volume estimate graph shows how the enclosed volume varies with the contour level. The recommended contour level is shown as a vertical line and the intersection between the line and the curve gives the volume of the enclosed surface at the given level.

CONFIDENTIAL REPORT

7.3 Rotationally averaged power spectrum (i)

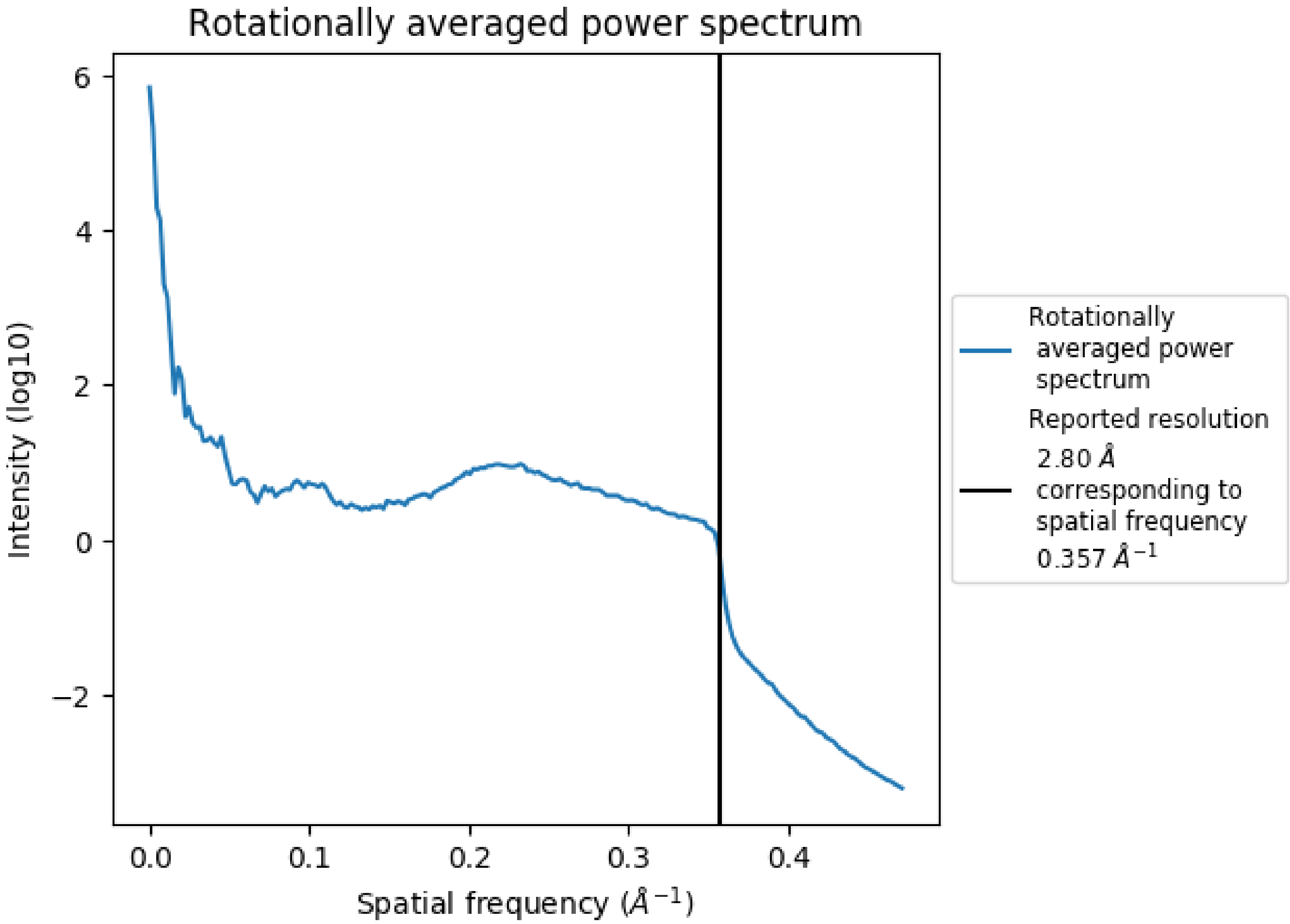

CONFIDENTIAL REPORT

8 Fourier-Shell correlation ⓘ

This section was not generated. No FSC curve or half maps provided.

CONFIDENTIAL VALIDATION REPORT

9 Map-model fit (i)

This section contains information regarding the fit between EMDB map EMD-11166 and PDB model 6ZCL. Per-residue inclusion information can be found in section 3 on page 5.

9.1 Map-model overlay (i)

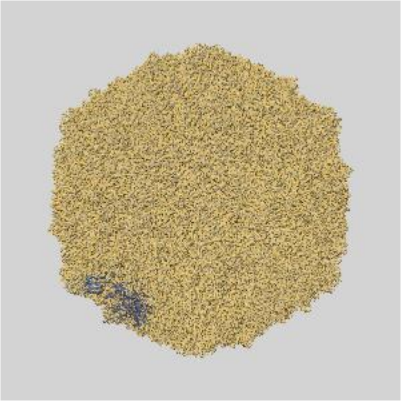

X

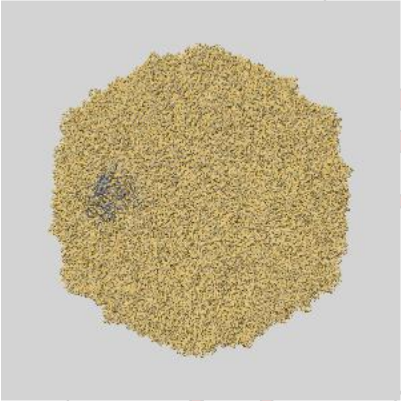

Y

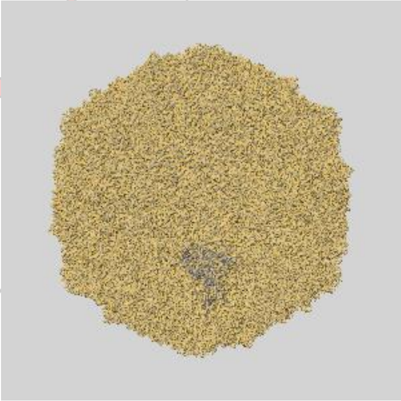

Z

The images above show the 3D surface view of the map at the recommended contour level 0.019 at 50% transparency in yellow overlaid with a ribbon representation of the model coloured in blue. These images allow for the visual assessment of the quality of fit between the atomic model and the map.

CONFIDENTIAL VALIDATION REPORT

9.2 Atom inclusion (i)

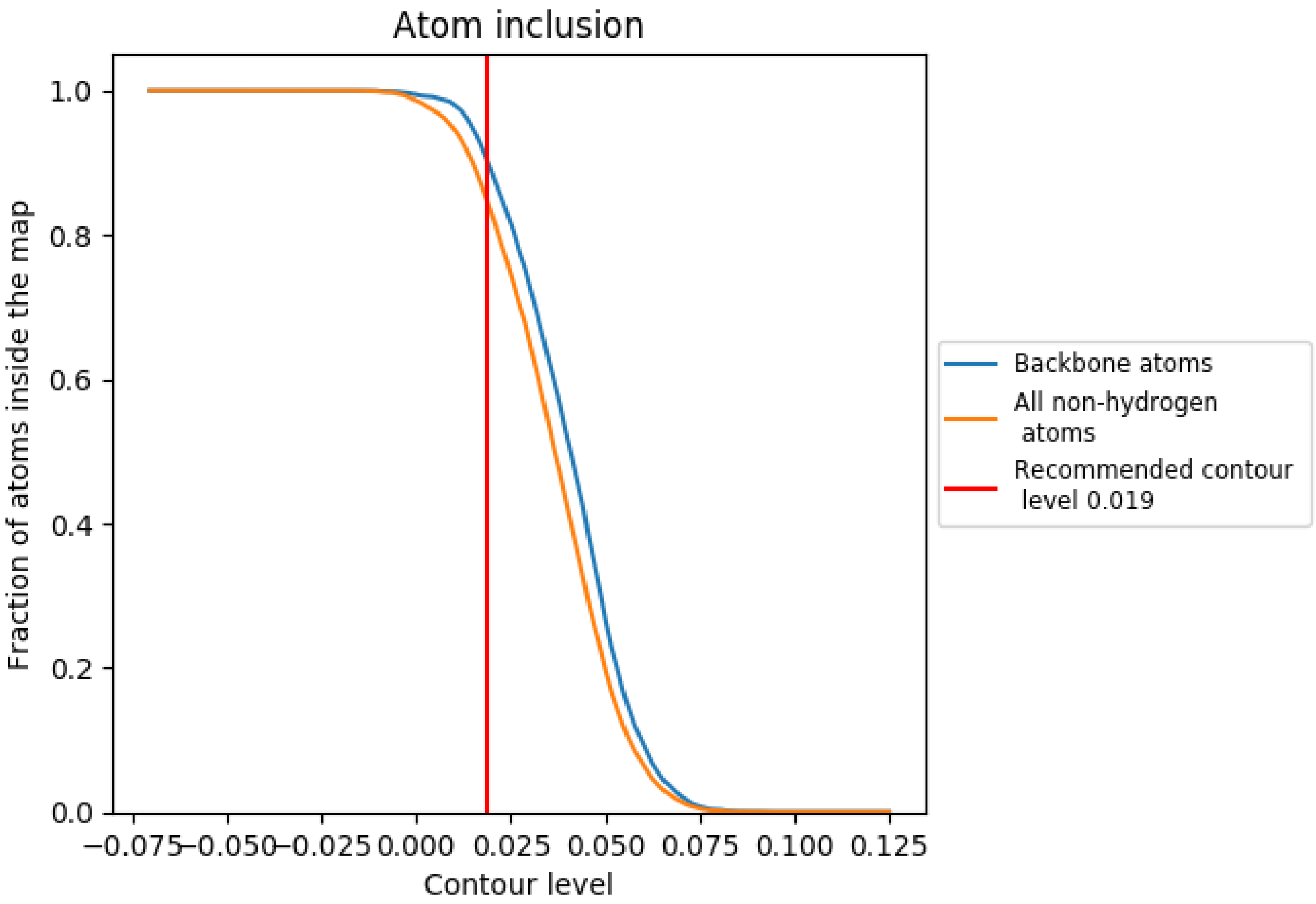

At the recommended contour level, 90% of all backbone atoms, 84% of all non-hydrogen atoms, are inside the map.

CONFIDENTIAL REPORT

Full wwPDB EM Map/Model Validation Report (i)

Jul 16, 2020 – 01:42 PM BST

PDB ID	:	6ZMS
EMDB ID	:	EMD-11300
Title	:	Coxsackievirus B4 strain E2
Deposited on	:	2020-07-03
Resolution	:	3.40 Å(reported)

This is a Full wwPDB EM Map/Model Validation Report.

This report is produced by the wwPDB biocuration pipeline after annotation of the structure.

We welcome your comments at *validation@mail.wwpdb.org*
A user guide is available at
https://www.wwpdb.org/validation/2017/EMValidationReportHelp
with specific help available everywhere you see the (i) symbol.

The following versions of software and data (see references (i)) were used in the production of this report:

EMDB validation analysis	:	0.0.0.dev33
MolProbity	:	4.02b-467
Percentile statistics	:	20191225.v01 (using entries in the PDB archive December 25th 2019)
Ideal geometry (proteins)	:	Engh & Huber (2001)
Ideal geometry (DNA, RNA)	:	Parkinson et al. (1996)
Validation Pipeline (wwPDB-VP)	:	2.13.dev1

CONFIDENTIAL VALIDATION REPORT

1 Overall quality at a glance (i)

The following experimental techniques were used to determine the structure:
ELECTRON MICROSCOPY

The reported resolution of this entry is 3.40 Å.

Percentile scores (ranging between 0-100) for global validation metrics of the entry are shown in the following graphic. The table shows the number of entries on which the scores are based.

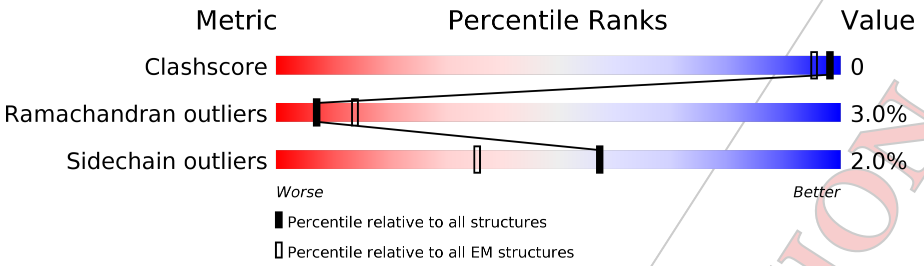

Metric	Whole archive (#Entries)	EM structures (#Entries)
Clashscore	158937	4297
Ramachandran outliers	154571	4023
Sidechain outliers	154315	3826

The table below summarises the geometric issues observed across the polymeric chains and their fit to the map. The red, orange, yellow and green segments on the bar indicate the fraction of residues that contain outliers for >=3, 2, 1 and 0 types of geometric quality criteria respectively. A grey segment represents the fraction of residues that are not modelled. The numeric value for each fraction is indicated below the corresponding segment, with a dot representing fractions <=5% The upper red bar (where present) indicates the fraction of residues that have poor fit to the EM map (all atom inclusion < 40%). The numeric value is given above the bar.

Mol	Chain	Length	Quality of chain
1	A	271	• 96% •
2	B	252	94% 5% •
3	C	238	• 95% • •
4	D	69	80% • 17%

CONFIDENTIAL VALIDATION REPORT

2 Entry composition (i)

There are 4 unique types of molecules in this entry. The entry contains 12570 atoms, of which 6173 are hydrogens and 0 are deuteriums.

In the tables below, the AltConf column contains the number of residues with at least one atom in alternate conformation and the Trace column contains the number of residues modelled with at most 2 atoms.

- Molecule 1 is a protein called Genome polyprotein.

Mol	Chain	Residues	Atoms						AltConf	Trace
			Total	C	H	N	O	S		
1	A	271	4236	1348	2083	379	411	15	0	0

- Molecule 2 is a protein called Genome polyprotein.

Mol	Chain	Residues	Atoms						AltConf	Trace
			Total	C	H	N	O	S		
2	B	252	3782	1221	1841	326	376	18	0	0

- Molecule 3 is a protein called Genome polyprotein.

Mol	Chain	Residues	Atoms						AltConf	Trace
			Total	C	H	N	O	S		
3	C	238	3661	1182	1808	304	350	17	0	0

- Molecule 4 is a protein called Genome polyprotein.

Mol	Chain	Residues	Atoms						AltConf	Trace
			Total	C	H	N	O	S		
4	D	57	891	280	441	78	91	1	0	0

CONFIDENTIAL VALIDATION REPORT

3 Residue-property plots (i)

These plots are drawn for all protein, RNA, DNA and oligosaccharide chains in the entry. The first graphic for a chain summarises the proportions of the various outlier classes displayed in the second graphic. The second graphic shows the sequence view annotated by issues in geometry and atom inclusion in map density. Residues are color-coded according to the number of geometric quality criteria for which they contain at least one outlier: green = 0, yellow = 1, orange = 2 and red = 3 or more. A red diamond above a residue indicates a poor fit to the EM map for this residue (all atom inclusion < 40%). Stretches of 2 or more consecutive residues without any outlier are shown as a green connector. Residues present in the sample, but not in the model, are shown in grey.

- Molecule 1: Genome polyprotein

Chain A:

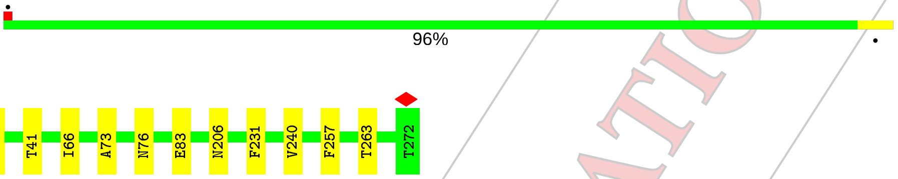

- Molecule 2: Genome polyprotein

Chain B:

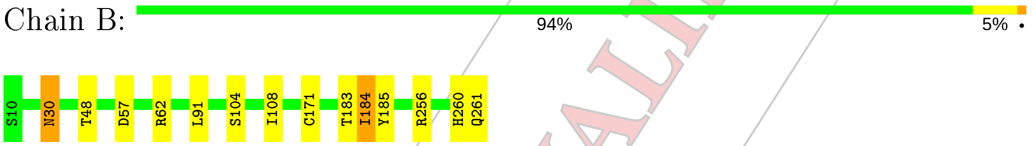

- Molecule 3: Genome polyprotein

Chain C:

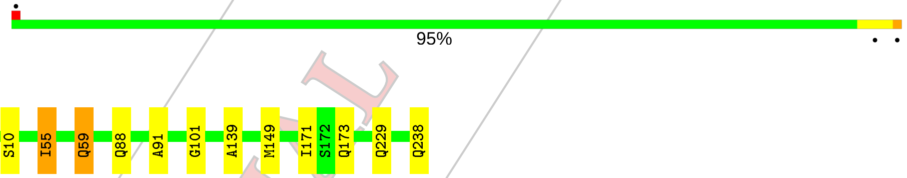

- Molecule 4: Genome polyprotein

Chain D:

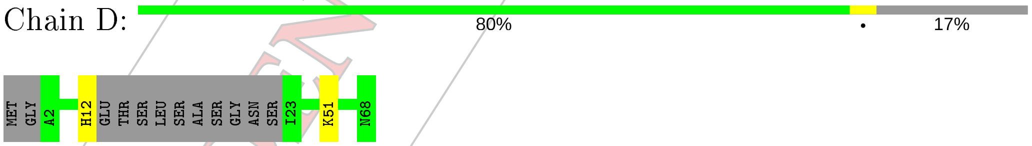

CONFIDENTIAL VALIDATION REPORT

4 Experimental information (i)

Property	Value	Source
EM reconstruction method	SINGLE PARTICLE	Depositor
Imposed symmetry	POINT, Not provided	Depositor
Number of particles used	40627	Depositor
Resolution determination method	FSC 0.143 CUT-OFF	Depositor
CTF correction method	PHASE FLIPPING AND AMPLITUDE CORRECTION	Depositor
Microscope	TFS TALOS F200C	Depositor
Voltage (kV)	200	Depositor
Electron dose $(e^-/\text{Å}^2)$	40	Depositor
Minimum defocus (nm)	Not provided	Depositor
Maximum defocus (nm)	Not provided	Depositor
Magnification	Not provided	Depositor
Image detector	FEI FALCON III (4k x 4k)	Depositor
Maximum map value	0.083	Depositor
Minimum map value	-0.046	Depositor
Average map value	0.001	Depositor
Map value standard deviation	0.005	Depositor
Recommended contour level	0.00816	Depositor
Map size (Å)	496.0, 496.0, 496.0	Depositor
Map dimensions	400, 400, 400	Depositor
Map angles (°)	90.0, 90.0, 90.0	Depositor
Pixel spacing (Å)	1.24, 1.24, 1.24	Depositor

CONFIDENTIAL VALIDATION REPORT

5 Model quality (i)

5.1 Standard geometry (i)

The Z score for a bond length (or angle) is the number of standard deviations the observed value is removed from the expected value. A bond length (or angle) with $|Z| > 5$ is considered an outlier worth inspection. RMSZ is the root-mean-square of all Z scores of the bond lengths (or angles).

Mol	Chain	Bond lengths		Bond angles	
		RMSZ	#$\|Z\|$ >5	RMSZ	#$\|Z\|$ >5
1	A	0.91	0/2209	1.04	2/3006 (0.1%)
2	B	0.90	0/1991	1.00	0/2720
3	C	0.90	0/1900	1.00	0/2585
4	D	0.86	0/457	0.97	0/614
All	All	0.90	0/6557	1.01	2/8925 (0.0%)

Chiral center outliers are detected by calculating the chiral volume of a chiral center and verifying if the center is modelled as a planar moiety or with the opposite hand.A planarity outlier is detected by checking planarity of atoms in a peptide group, atoms in a mainchain group or atoms of a sidechain that are expected to be planar.

Mol	Chain	#Chirality outliers	#Planarity outliers
2	B	0	1
4	D	0	1
All	All	0	2

There are no bond length outliers.

All (2) bond angle outliers are listed below:

Mol	Chain	Res	Type	Atoms	Z	Observed(^{o})	Ideal(^{o})
1	A	231	PHE	CB-CG-CD2	-5.57	116.90	120.80
1	A	231	PHE	CB-CG-CD1	5.55	124.69	120.80

There are no chirality outliers.

All (2) planarity outliers are listed below:

Mol	Chain	Res	Type	Group
2	B	185	TYR	Sidechain
4	D	12	HIS	Sidechain

CONFIDENTIAL VALIDATION REPORT

5.2 Too-close contacts (i)

In the following table, the Non-H and H(model) columns list the number of non-hydrogen atoms and hydrogen atoms in the chain respectively. The H(added) column lists the number of hydrogen atoms added and optimized by MolProbity. The Clashes column lists the number of clashes within the asymmetric unit, whereas Symm-Clashes lists symmetry related clashes.

Mol	Chain	Non-H	H(model)	H(added)	Clashes	Symm-Clashes
1	A	2153	2083	2080	1	0
2	B	1941	1841	1838	2	0
3	C	1853	1808	1808	1	0
4	D	450	441	434	0	0
All	All	6397	6173	6160	4	0

The all-atom clashscore is defined as the number of clashes found per 1000 atoms (including hydrogen atoms). The all-atom clashscore for this structure is 0.

All (4) close contacts within the same asymmetric unit are listed below, sorted by their clash magnitude.

Atom-1	Atom-2	Interatomic distance (Å)	Clash overlap (Å)
2:B:108:ILE:H	2:B:108:ILE:HD12	1.66	0.61
1:A:19:ILE:H	1:A:19:ILE:HD13	1.81	0.46
2:B:184:ILE:HD12	2:B:184:ILE:H	1.84	0.42
3:C:55:ILE:H	3:C:55:ILE:HD13	1.84	0.42

There are no symmetry-related clashes.

5.3 Torsion angles (i)

5.3.1 Protein backbone (i)

In the following table, the Percentiles column shows the percent Ramachandran outliers of the chain as a percentile score with respect to all PDB entries followed by that with respect to all EM entries.

The Analysed column shows the number of residues for which the backbone conformation was analysed, and the total number of residues.

Mol	Chain	Analysed	Favoured	Allowed	Outliers	Percentiles	
1	A	269/271 (99%)	246 (91%)	15 (6%)	8 (3%)	4	23
2	B	250/252 (99%)	229 (92%)	13 (5%)	8 (3%)	4	22
3	C	236/238 (99%)	215 (91%)	14 (6%)	7 (3%)	4	23

Continued on next page...

CONFIDENTIAL VALIDATION REPORT

Continued from previous page...

Mol	Chain	Analysed	Favoured	Allowed	Outliers	Percentiles	
4	D	51/69 (74%)	47 (92%)	3 (6%)	1 (2%)	7	30
All	All	806/830 (97%)	737 (91%)	45 (6%)	24 (3%)	7	23

All (24) Ramachandran outliers are listed below:

Mol	Chain	Res	Type
2	B	171	CYS
3	C	88	GLN
1	A	66	ILE
1	A	76	ASN
1	A	206	ASN
2	B	30	ASN
2	B	260	HIS
1	A	41	THR
1	A	73	ALA
2	B	57	ASP
2	B	62	ARG
3	C	59	GLN
3	C	91	ALA
3	C	101	GLY
3	C	139	ALA
4	D	51	LYS
1	A	240	VAL
1	A	257	PHE
3	C	10	SER
2	B	48	THR
2	B	104	SER
2	B	183	THR
1	A	83	GLU
3	C	171	ILE

5.3.2 Protein sidechains (i)

In the following table, the Percentiles column shows the percent sidechain outliers of the chain as a percentile score with respect to all PDB entries followed by that with respect to all EM entries.

The Analysed column shows the number of residues for which the sidechain conformation was analysed, and the total number of residues.

CONFIDENTIAL VALIDATION REPORT

Mol	Chain	Analysed	Rotameric	Outliers	Percentiles	
1	A	242/242 (100%)	239 (99%)	3 (1%)	71	85
2	B	210/210 (100%)	205 (98%)	5 (2%)	49	74
3	C	207/207 (100%)	201 (97%)	6 (3%)	42	69
4	D	50/59 (85%)	50 (100%)	0	100	100
All	All	709/718 (99%)	695 (98%)	14 (2%)	57	77

All (14) residues with a non-rotameric sidechain are listed below:

Mol	Chain	Res	Type
1	A	19	ILE
1	A	27	THR
1	A	263	THR
2	B	30	ASN
2	B	91	LEU
2	B	184	ILE
2	B	256	ARG
2	B	261	GLN
3	C	55	ILE
3	C	59	GLN
3	C	149	MET
3	C	173	GLN
3	C	229	GLN
3	C	238	GLN

Some sidechains can be flipped to improve hydrogen bonding and reduce clashes. All (7) such sidechains are listed below:

Mol	Chain	Res	Type
1	A	131	GLN
1	A	214	HIS
1	A	248	GLN
2	B	167	GLN
2	B	206	ASN
3	C	20	GLN
4	D	12	HIS

5.3.3 RNA (i)

There are no RNA molecules in this entry.

CONFIDENTIAL VALIDATION REPORT

5.4 Non-standard residues in protein, DNA, RNA chains (i)

There are no non-standard protein/DNA/RNA residues in this entry.

5.5 Carbohydrates (i)

There are no carbohydrates in this entry.

5.6 Ligand geometry (i)

There are no ligands in this entry.

5.7 Other polymers (i)

There are no such residues in this entry.

5.8 Polymer linkage issues (i)

The following chains have linkage breaks:

Mol	Chain	Number of breaks
4	D	1

All chain breaks are listed below:

Model	Chain	Residue-1	Atom-1	Residue-2	Atom-2	Distance (Å)
1	D	10:GLY	C	11:ALA	N	3.36

CONFIDENTIAL VALIDATION REPORT

6 Map visualisation ⓘ

This section contains visualisations of the EMDB entry EMD-11300. These are intended to permit visual inspection of the internal detail of the map and identification of artifacts.

6.1 Orthogonal projections ⓘ

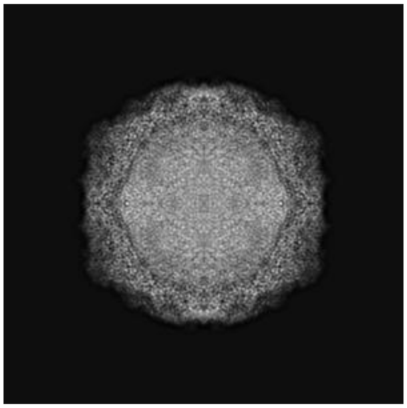

X

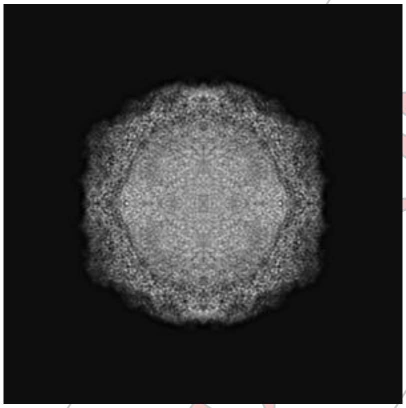

Y

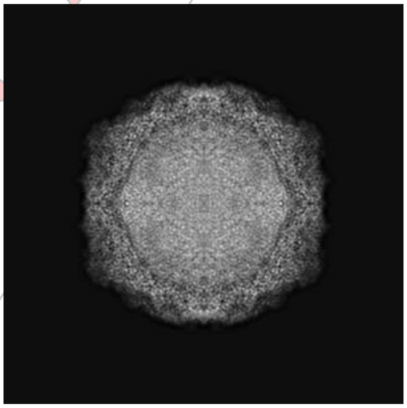

Z

The images above show the map projected in three orthogonal projections, in greyscale.

6.2 Central slices ⓘ

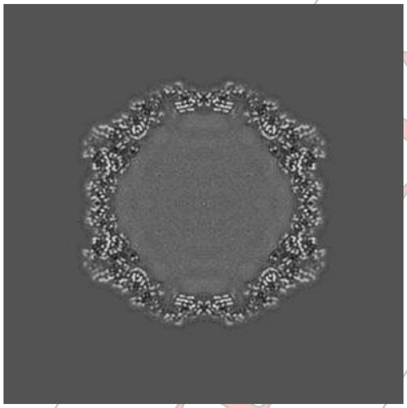

X

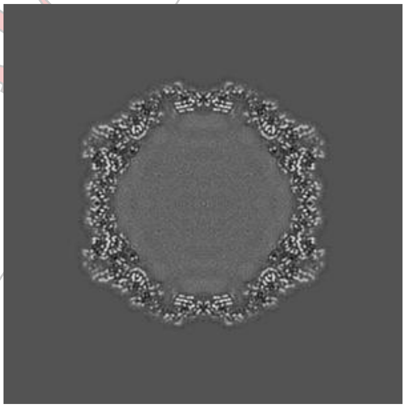

Y

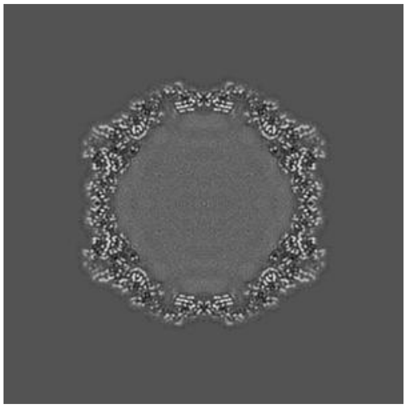

Z

The images above show central slices of the map in three orthogonal directions, in greyscale.

CONFIDENTIAL VALIDATION REPORT

6.3 Largest variance slices (i)

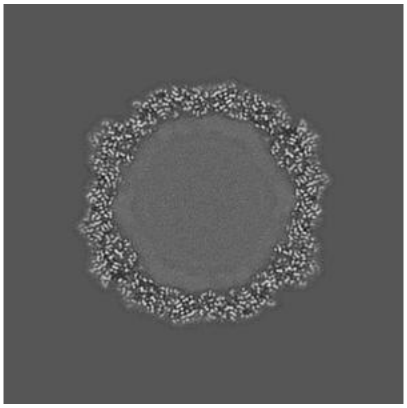

X Index: 212

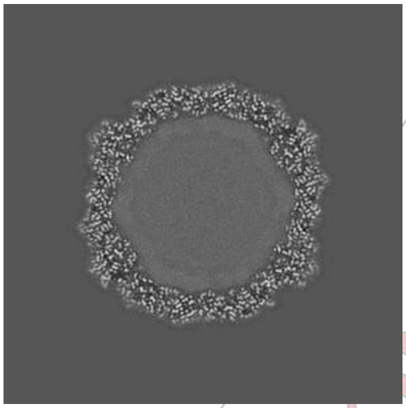

Y Index: 212

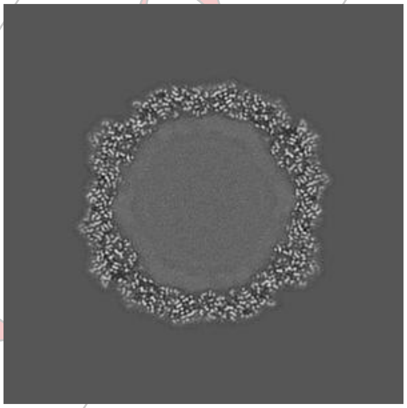

Z Index: 212

The images above show the highest variance slices of the map in three orthogonal directions, in greyscale.

6.4 Orthogonal surface views (i)

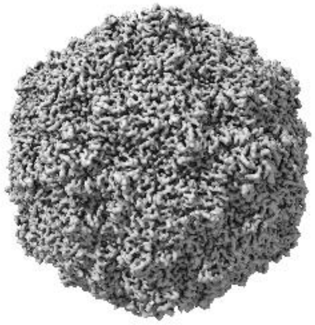

X

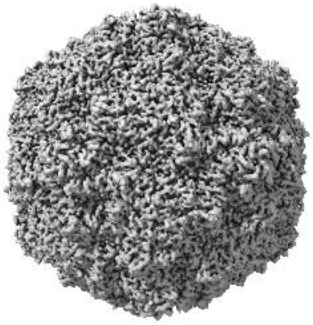

Y

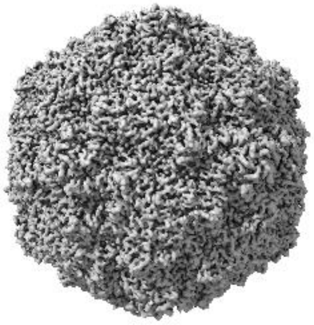

Z

The images above show the 3D surface view of the map at the recommended contour level 0.00816. This in conjunction with the slice images can indicate whether an appropriate contour level has been selected.

6.5 Mask visualisation (i)

This section was not generated. No masks were provided.

CONFIDENTIAL REPORT

7 Map analysis (i)

This section contains the results of statistical analysis of the map.

7.1 Map-value distribution (i)

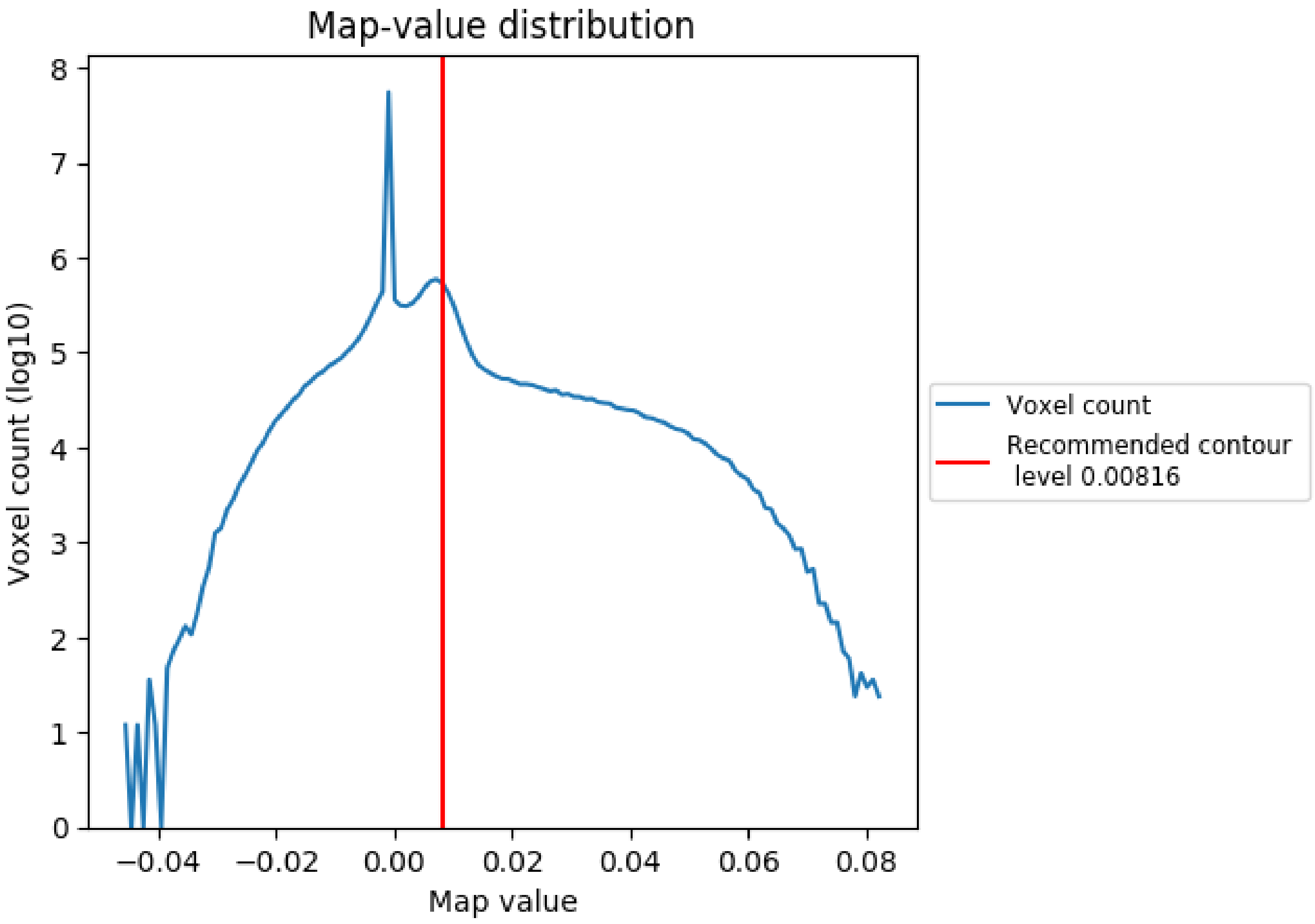

The map-value distribution is plotted in 128 intervals along the x-axis. The y-axis is logarithmic. A spike in this graph at zero usually indicates that the volume has been masked.

7.2 Volume estimate ⓘ

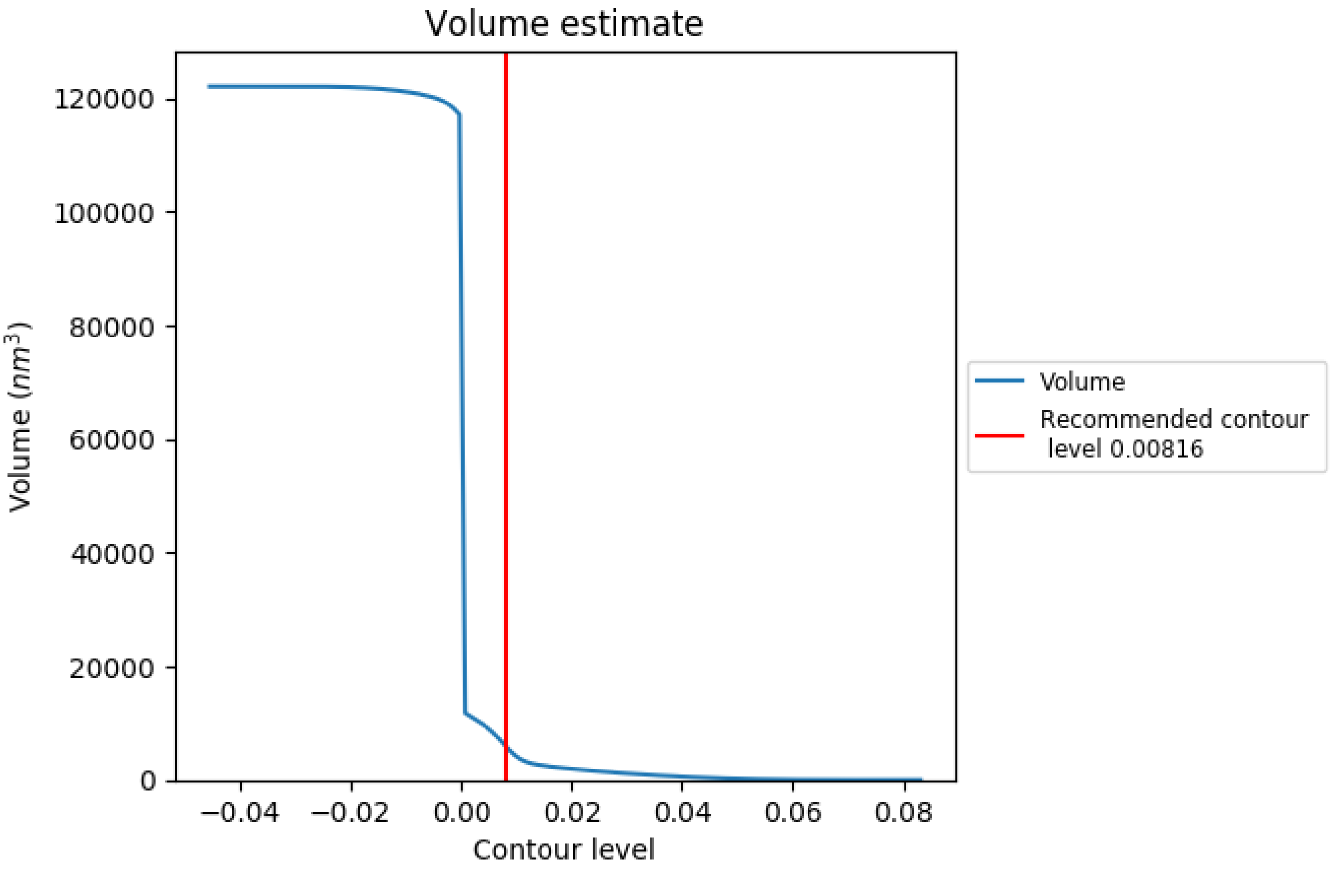

The volume at the recommended contour level is 5872 nm^3; this corresponds to an approximate mass of 5304 kDa.

The volume estimate graph shows how the enclosed volume varies with the contour level. The recommended contour level is shown as a vertical line and the intersection between the line and the curve gives the volume of the enclosed surface at the given level.

CONFIDENTIAL REPORT

7.3 Rotationally averaged power spectrum ⓘ

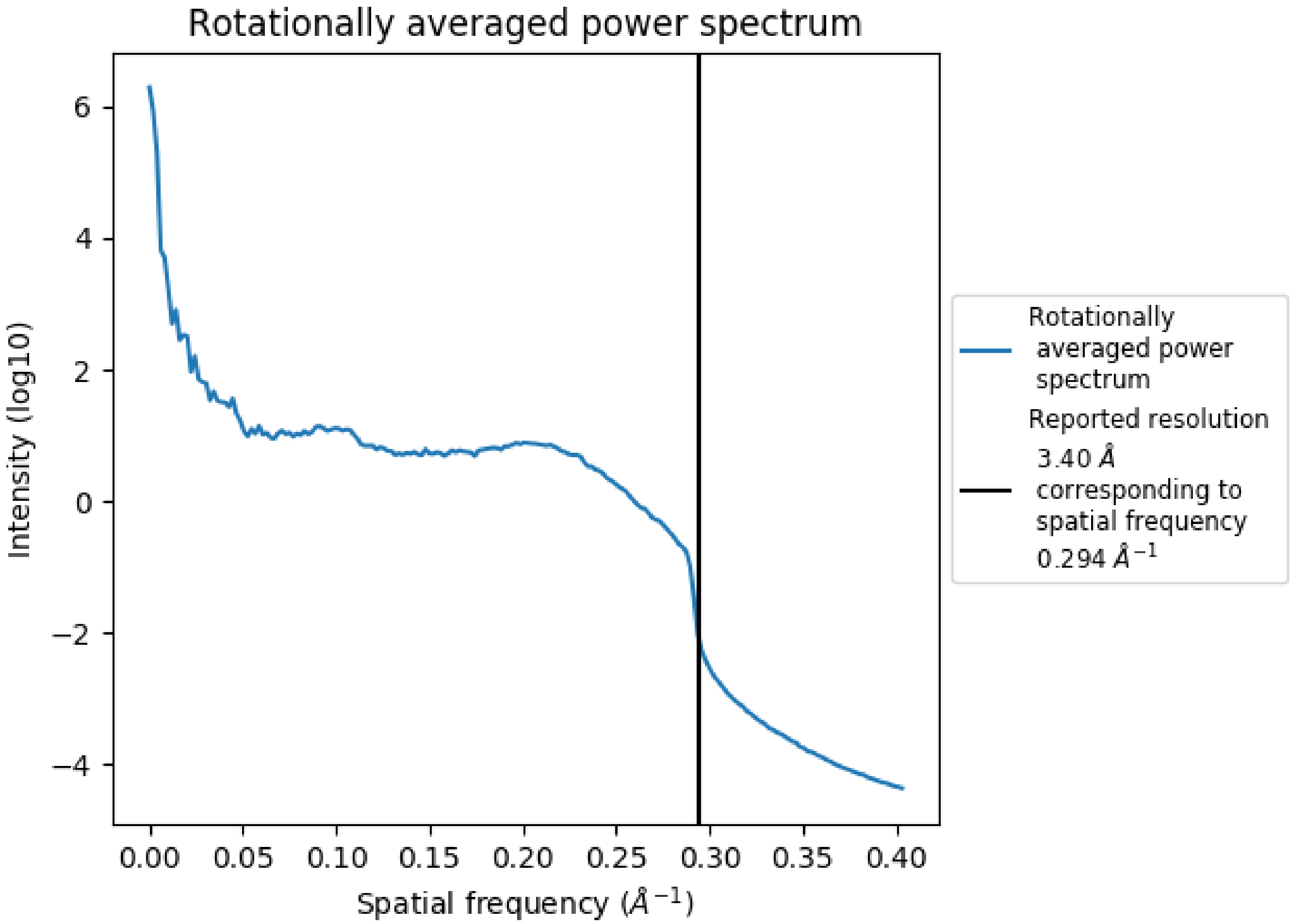

CONFIDENTIAL

8 Fourier-Shell correlation (i)

This section was not generated. No FSC curve or half maps provided.

CONFIDENTIAL VALIDATION REPORT

9 Map-model fit (i)

This section contains information regarding the fit between EMDB map EMD-11300 and PDB model 6ZMS. Per-residue inclusion information can be found in section 3 on page 4.

9.1 Map-model overlay (i)

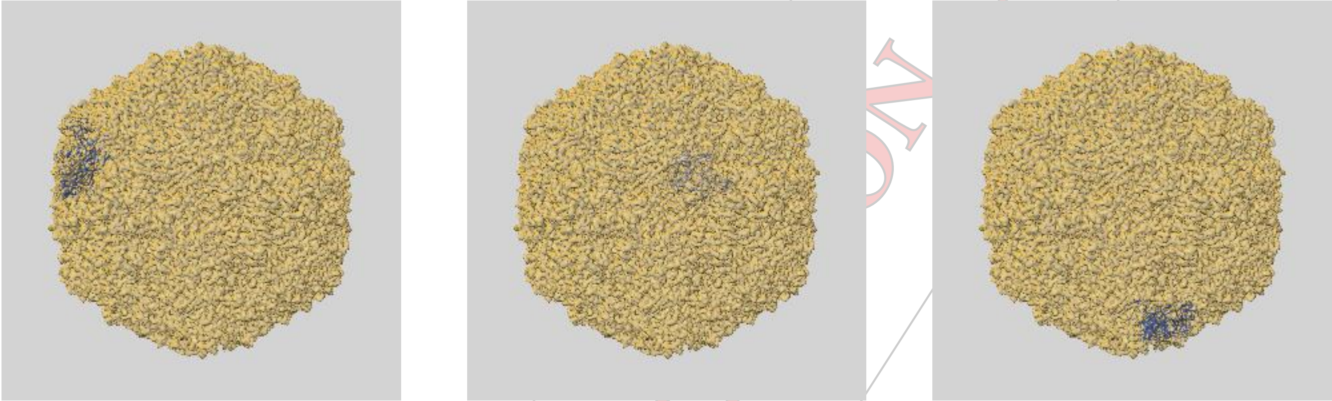

X Y Z

The images above show the 3D surface view of the map at the recommended contour level 0.00816 at 50% transparency in yellow overlaid with a ribbon representation of the model coloured in blue. These images allow for the visual assessment of the quality of fit between the atomic model and the map.

CONFIDENTIAL VALIDATION REPORT

9.2 Atom inclusion (i)

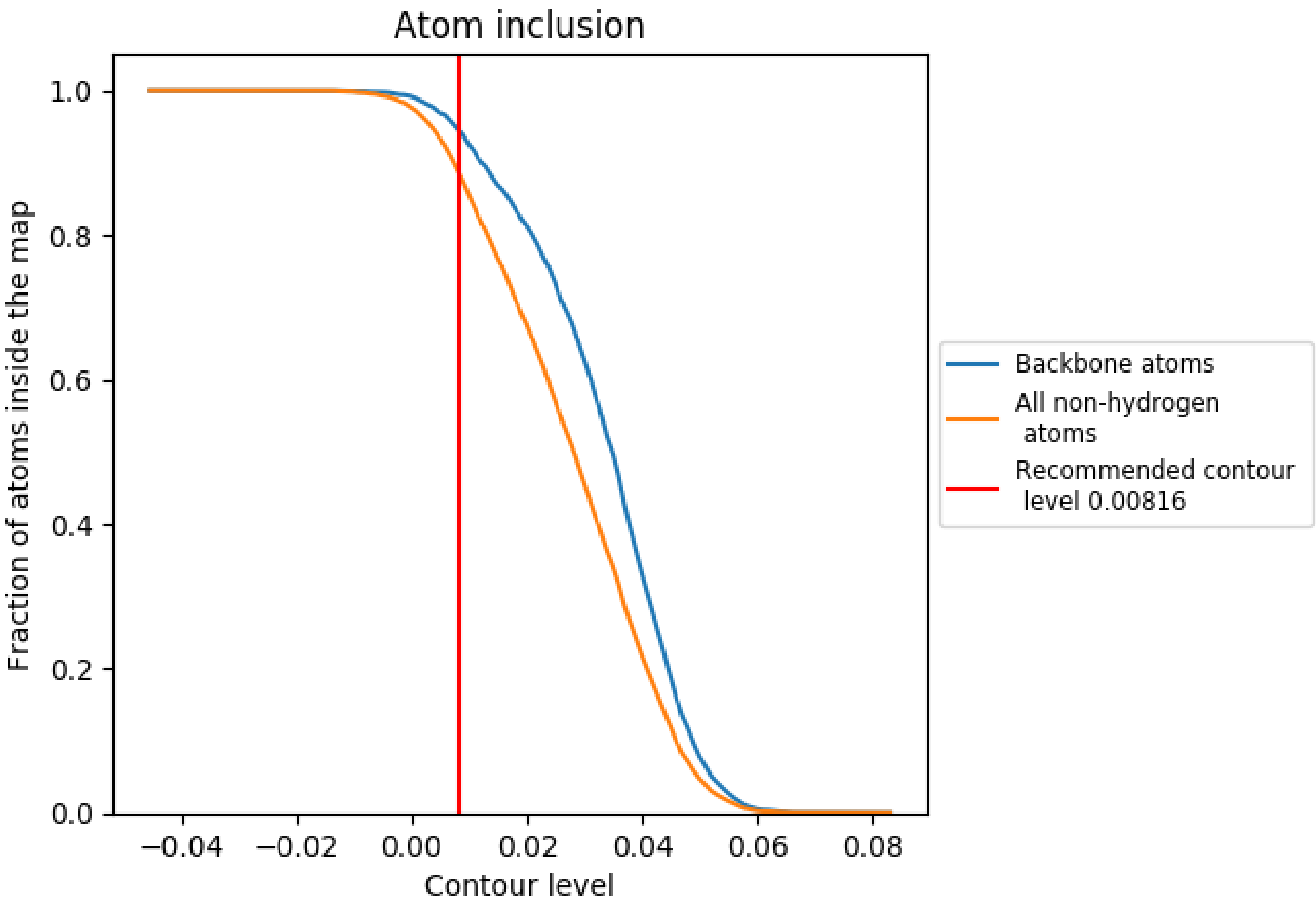

At the recommended contour level, 95% of all backbone atoms, 89% of all non-hydrogen atoms, are inside the map.

CONFIDENTIAL REPORT

Full wwPDB EM Map/Model Validation Report (i)

Jul 3, 2020 – 11:43 AM BST

PDB ID	:	6ZCK
EMDB ID	:	EMD-11165
Title	:	Coxsackievirus B4 in complex with capsid binder compound 48
Deposited on	:	2020-06-11
Resolution	:	2.70 Å(reported)

This is a Full wwPDB EM Map/Model Validation Report.

This report is produced by the wwPDB biocuration pipeline after annotation of the structure.

We welcome your comments at *validation@mail.wwpdb.org*
A user guide is available at
https://www.wwpdb.org/validation/2017/EMValidationReportHelp
with specific help available everywhere you see the (i) symbol.

CONFIDENTIAL VALIDATION REPORT

The following versions of software and data (see references (i)) were used in the production of this report:

EMDB validation analysis	:	0.0.0.dev33
Mogul	:	1.8.5 (274361), CSD as541be (2020)
MolProbity	:	4.02b-467
buster-report	:	1.1.7 (2018)
Percentile statistics	:	20191225.v01 (using entries in the PDB archive December 25th 2019)
Ideal geometry (proteins)	:	Engh & Huber (2001)
Ideal geometry (DNA, RNA)	:	Parkinson et al. (1996)
Validation Pipeline (wwPDB-VP)	:	2.13.dev1

1 Overall quality at a glance (i)

The following experimental techniques were used to determine the structure:
ELECTRON MICROSCOPY

The reported resolution of this entry is 2.70 Å.

Percentile scores (ranging between 0-100) for global validation metrics of the entry are shown in the following graphic. The table shows the number of entries on which the scores are based.

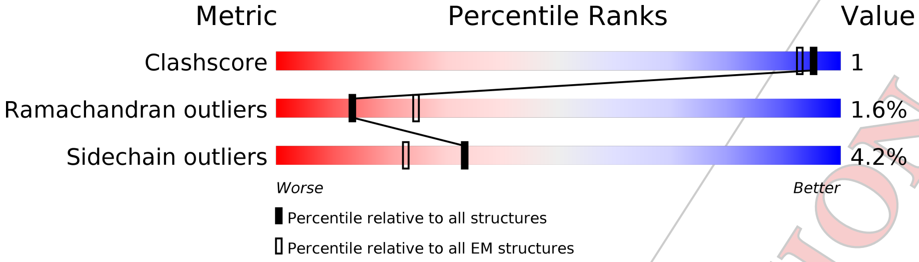

Metric	Whole archive (#Entries)	EM structures (#Entries)
Clashscore	158937	4297
Ramachandran outliers	154571	4023
Sidechain outliers	154315	3826

The table below summarises the geometric issues observed across the polymeric chains and their fit to the map. The red, orange, yellow and green segments on the bar indicate the fraction of residues that contain outliers for >=3, 2, 1 and 0 types of geometric quality criteria respectively. A grey segment represents the fraction of residues that are not modelled. The numeric value for each fraction is indicated below the corresponding segment, with a dot representing fractions <=5% The upper red bar (where present) indicates the fraction of residues that have poor fit to the EM map (all atom inclusion < 40%). The numeric value is given above the bar.

Mol	Chain	Length	Quality of chain
1	A	271	93% 6% •
2	B	252	93% 6% •
3	C	238	87% 13%
4	D	58	• 93% 7%

CONFIDENTIAL VALIDATION REPORT

2 Entry composition (i)

There are 6 unique types of molecules in this entry. The entry contains 12628 atoms, of which 6185 are hydrogens and 0 are deuteriums.

In the tables below, the AltConf column contains the number of residues with at least one atom in alternate conformation and the Trace column contains the number of residues modelled with at most 2 atoms.

- Molecule 1 is a protein called Genome polyprotein.

Mol	Chain	Residues	Atoms						AltConf	Trace
			Total	C	H	N	O	S		
1	A	271	4234	1348	2081	379	411	15	0	0

- Molecule 2 is a protein called Genome polyprotein.

Mol	Chain	Residues	Atoms						AltConf	Trace
			Total	C	H	N	O	S		
2	B	252	3782	1221	1841	326	376	18	0	0

- Molecule 3 is a protein called Genome polyprotein.

Mol	Chain	Residues	Atoms						AltConf	Trace
			Total	C	H	N	O	S		
3	C	238	3661	1182	1808	304	350	17	0	0

- Molecule 4 is a protein called Genome polyprotein.

Mol	Chain	Residues	Atoms						AltConf	Trace
			Total	C	H	N	O	S		
4	D	58	889	282	436	79	91	1	0	0

There are 10 discrepancies between the modelled and reference sequences:

Chain	Residue	Modelled	Actual	Comment	Reference
D	?	-	GLU	deletion	UNP Q8V639
D	?	-	THR	deletion	UNP Q8V639
D	?	-	SER	deletion	UNP Q8V639
D	?	-	LEU	deletion	UNP Q8V639
D	?	-	SER	deletion	UNP Q8V639
D	?	-	ALA	deletion	UNP Q8V639
D	?	-	SER	deletion	UNP Q8V639
D	?	-	GLY	deletion	UNP Q8V639
D	?	-	ASN	deletion	UNP Q8V639

Continued on next page...

CONFIDENTIAL VALIDATION REPORT

Continued from previous page...

Chain	Residue	Modelled	Actual	Comment	Reference
D	?	-	SER	deletion	UNP Q8V639

- Molecule 5 is 2-oxidanyl-4-[(6-propoxynaphthalen-2-yl)sulfonylamino]benzoic acid (three-letter code: QFW) (formula: $C_{20}H_{19}NO_6S$) (labeled as "Ligand of Interest" by author).

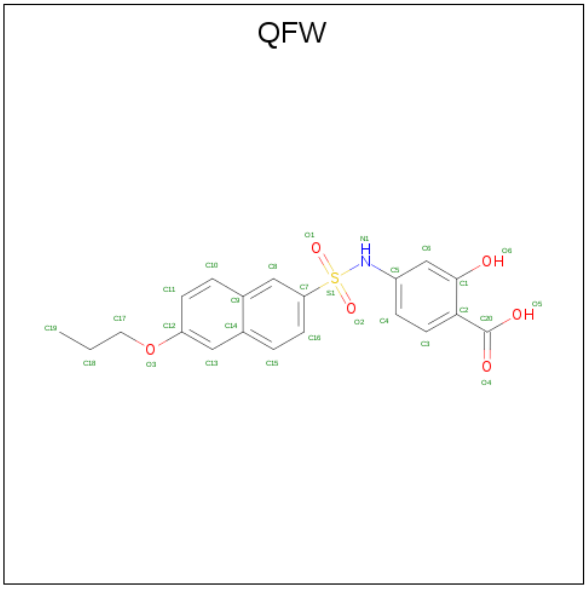

Mol	Chain	Residues	Atoms						AltConf
			Total	C	H	N	O	S	
5	C	1	47	20	19	1	6	1	0

- Molecule 6 is MYRISTIC ACID (three-letter code: MYR) (formula: $C_{14}H_{28}O_2$).

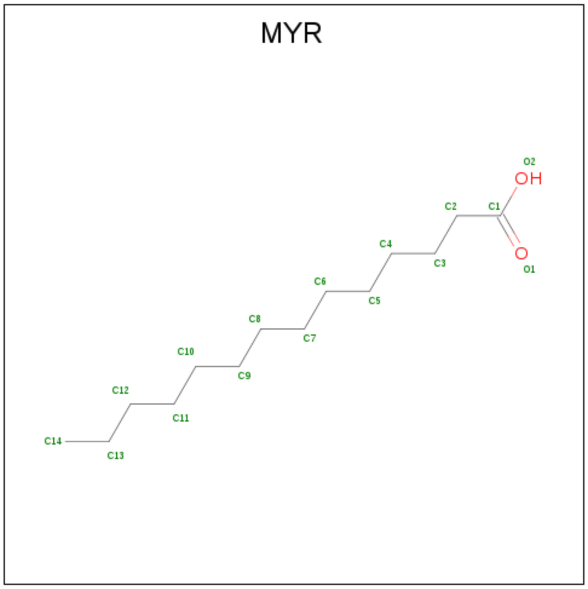

Mol	Chain	Residues	Atoms			AltConf
			Total	C	O	
6	D	1	15	14	1	0

CONFIDENTIAL VALIDATION REPORT

3 Residue-property plots (i)

These plots are drawn for all protein, RNA, DNA and oligosaccharide chains in the entry. The first graphic for a chain summarises the proportions of the various outlier classes displayed in the second graphic. The second graphic shows the sequence view annotated by issues in geometry and atom inclusion in map density. Residues are color-coded according to the number of geometric quality criteria for which they contain at least one outlier: green = 0, yellow = 1, orange = 2 and red = 3 or more. A red diamond above a residue indicates a poor fit to the EM map for this residue (all atom inclusion < 40%). Stretches of 2 or more consecutive residues without any outlier are shown as a green connector. Residues present in the sample, but not in the model, are shown in grey.

- Molecule 1: Genome polyprotein

Chain A:

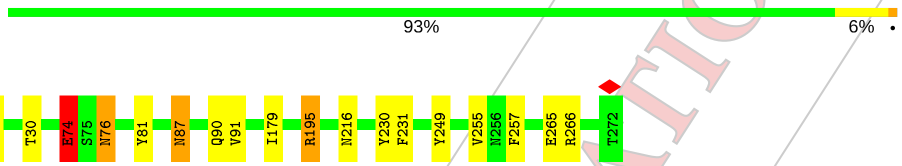

- Molecule 2: Genome polyprotein

Chain B:

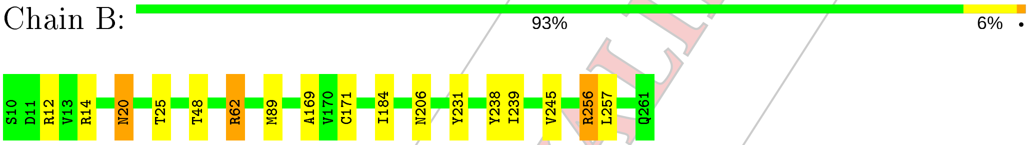

- Molecule 3: Genome polyprotein

Chain C:

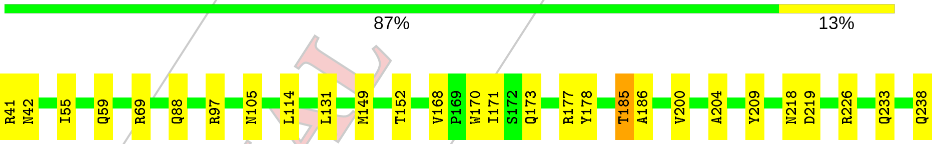

- Molecule 4: Genome polyprotein

Chain D:

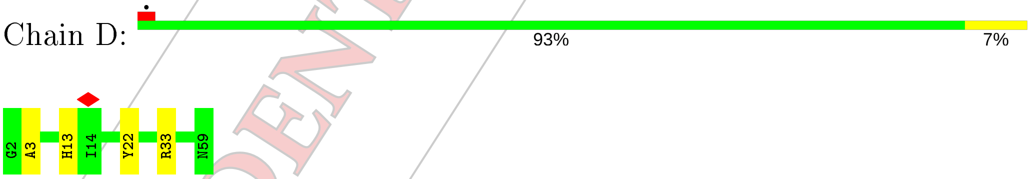

CONFIDENTIAL VALIDATION REPORT

4 Experimental information ⓘ

Property	Value	Source
EM reconstruction method	SINGLE PARTICLE	Depositor
Imposed symmetry	POINT, Not provided	Depositor
Number of particles used	13252	Depositor
Resolution determination method	FSC 0.143 CUT-OFF	Depositor
CTF correction method	PHASE FLIPPING AND AMPLITUDE CORRECTION	Depositor
Microscope	FEI TITAN KRIOS	Depositor
Voltage (kV)	300	Depositor
Electron dose $(e^-/\text{Å}^2)$	47	Depositor
Minimum defocus (nm)	Not provided	Depositor
Maximum defocus (nm)	Not provided	Depositor
Magnification	Not provided	Depositor
Image detector	GATAN K2 SUMMIT (4k x 4k)	Depositor
Maximum map value	0.078	Depositor
Minimum map value	-0.035	Depositor
Average map value	0.001	Depositor
Map value standard deviation	0.006	Depositor
Recommended contour level	0.00928	Depositor
Map size (Å)	445.19998, 445.19998, 445.19998	Depositor
Map dimensions	420, 420, 420	Depositor
Map angles (°)	90.0, 90.0, 90.0	Depositor
Pixel spacing (Å)	1.06, 1.06, 1.06	Depositor

CONFIDENTIAL VALIDATION REPORT

5 Model quality (i)

5.1 Standard geometry (i)

Bond lengths and bond angles in the following residue types are not validated in this section: QFW, MYR

The Z score for a bond length (or angle) is the number of standard deviations the observed value is removed from the expected value. A bond length (or angle) with $|Z| > 5$ is considered an outlier worth inspection. RMSZ is the root-mean-square of all Z scores of the bond lengths (or angles).

Mol	Chain	Bond lengths		Bond angles	
		RMSZ	#$|Z|$ >5	RMSZ	#$|Z|$ >5
1	A	0.91	0/2209	1.16	3/3006 (0.1%)
2	B	0.91	0/1991	1.10	2/2720 (0.1%)
3	C	0.92	0/1900	1.14	6/2585 (0.2%)
4	D	0.89	1/460 (0.2%)	1.02	0/619
All	All	0.91	1/6560 (0.0%)	1.13	11/8930 (0.1%)

Chiral center outliers are detected by calculating the chiral volume of a chiral center and verifying if the center is modelled as a planar moiety or with the opposite hand.A planarity outlier is detected by checking planarity of atoms in a peptide group, atoms in a mainchain group or atoms of a sidechain that are expected to be planar.

Mol	Chain	#Chirality outliers	#Planarity outliers
1	A	0	7
2	B	0	4
4	D	0	2
All	All	0	13

All (1) bond length outliers are listed below:

Mol	Chain	Res	Type	Atoms	Z	Observed(Å)	Ideal(Å)
4	D	3	ALA	N-CA	5.94	1.58	1.46

All (11) bond angle outliers are listed below:

Mol	Chain	Res	Type	Atoms	Z	Observed(^{o})	Ideal(^{o})
3	C	41	ARG	NE-CZ-NH1	7.64	124.12	120.30
1	A	74	GLU	N-CA-CB	6.51	122.31	110.60
3	C	34	MET	CG-SD-CE	-6.31	90.11	100.20
3	C	177	ARG	NE-CZ-NH1	5.97	123.29	120.30
3	C	209	TYR	CB-CG-CD1	-5.92	117.45	121.00

Continued on next page...

CONFIDENTIAL VALIDATION REPORT

Continued from previous page...

Mol	Chain	Res	Type	Atoms	Z	Observed(^{o})	Ideal(^{o})
1	A	231	PHE	CB-CG-CD1	5.88	124.92	120.80
3	C	97	ARG	NE-CZ-NH1	5.75	123.17	120.30
1	A	231	PHE	CB-CG-CD2	-5.74	116.78	120.80
2	B	62	ARG	NE-CZ-NH1	5.63	123.12	120.30
2	B	12	ARG	NE-CZ-NH2	-5.04	117.78	120.30
3	C	69	ARG	NE-CZ-NH1	5.01	122.81	120.30

There are no chirality outliers.

All (13) planarity outliers are listed below:

Mol	Chain	Res	Type	Group
1	A	11	ARG	Sidechain
1	A	195	ARG	Sidechain
1	A	230	TYR	Sidechain
1	A	249	TYR	Sidechain
1	A	255	VAL	Peptide
1	A	266	ARG	Sidechain
1	A	81	TYR	Sidechain
2	B	14	ARG	Sidechain
2	B	231	TYR	Sidechain
2	B	238	TYR	Sidechain
2	B	256	ARG	Sidechain
4	D	13	HIS	Sidechain
4	D	33	ARG	Sidechain

5.2 Too-close contacts ⓘ

In the following table, the Non-H and H(model) columns list the number of non-hydrogen atoms and hydrogen atoms in the chain respectively. The H(added) column lists the number of hydrogen atoms added and optimized by MolProbity. The Clashes column lists the number of clashes within the asymmetric unit, whereas Symm-Clashes lists symmetry related clashes.

Mol	Chain	Non-H	H(model)	H(added)	Clashes	Symm-Clashes
1	A	2153	2081	2080	3	0
2	B	1941	1841	1838	1	0
3	C	1853	1808	1808	6	0
4	D	453	436	437	5	0
5	C	28	19	0	0	0
6	D	15	0	27	4	0
All	All	6443	6185	6190	12	0

CONFIDENTIAL VALIDATION REPORT

The all-atom clashscore is defined as the number of clashes found per 1000 atoms (including hydrogen atoms). The all-atom clashscore for this structure is 1.

All (12) close contacts within the same asymmetric unit are listed below, sorted by their clash magnitude.

Atom-1	Atom-2	Interatomic distance (Å)	Clash overlap (Å)
4:D:22:TYR:HD1	6:D:101:MYR:H131	1.44	0.82
4:D:22:TYR:CD1	6:D:101:MYR:H131	2.28	0.66
2:B:20:ASN:HD22	2:B:20:ASN:H	1.41	0.65
4:D:22:TYR:CD1	6:D:101:MYR:C13	2.87	0.57
1:A:87:ASN:HD21	1:A:90:GLN:HE21	1.53	0.57
3:C:105:ASN:HD22	3:C:226:ARG:HH22	1.55	0.55
1:A:91:VAL:HA	3:C:233:GLN:HE22	1.75	0.51
3:C:20:GLN:HE22	4:D:22:TYR:H	1.58	0.51
4:D:22:TYR:HD1	6:D:101:MYR:C13	2.16	0.51
3:C:178:TYR:H	3:C:185:THR:HG21	1.78	0.49
3:C:200:VAL:HG22	3:C:204:ALA:HB3	1.95	0.47
1:A:5:VAL:HB	3:C:219:ASP:HA	2.04	0.40

There are no symmetry-related clashes.

5.3 Torsion angles ⓘ

5.3.1 Protein backbone ⓘ

In the following table, the Percentiles column shows the percent Ramachandran outliers of the chain as a percentile score with respect to all PDB entries followed by that with respect to all EM entries.

The Analysed column shows the number of residues for which the backbone conformation was analysed, and the total number of residues.

Mol	Chain	Analysed	Favoured	Allowed	Outliers	Percentiles	
1	A	269/271 (99%)	246 (91%)	18 (7%)	5 (2%)	8	20
2	B	250/252 (99%)	233 (93%)	13 (5%)	4 (2%)	9	24
3	C	236/238 (99%)	222 (94%)	10 (4%)	4 (2%)	9	23
4	D	52/58 (90%)	50 (96%)	2 (4%)	0	100	100
All	All	807/819 (98%)	751 (93%)	43 (5%)	13 (2%)	13	24

All (13) Ramachandran outliers are listed below:

CONFIDENTIAL VALIDATION REPORT

Mol	Chain	Res	Type
1	A	195	ARG
1	A	74	GLU
1	A	76	ASN
2	B	62	ARG
2	B	169	ALA
2	B	171	CYS
3	C	88	GLN
1	A	257	PHE
1	A	265	GLU
2	B	257	LEU
3	C	171	ILE
3	C	170	TRP
3	C	186	ALA

5.3.2 Protein sidechains (i)

In the following table, the Percentiles column shows the percent sidechain outliers of the chain as a percentile score with respect to all PDB entries followed by that with respect to all EM entries.

The Analysed column shows the number of residues for which the sidechain conformation was analysed, and the total number of residues.

Mol	Chain	Analysed	Rotameric	Outliers	Percentiles	
1	A	242/242 (100%)	234 (97%)	8 (3%)	38	67
2	B	210/210 (100%)	201 (96%)	9 (4%)	29	57
3	C	207/207 (100%)	194 (94%)	13 (6%)	18	40
4	D	50/50 (100%)	50 (100%)	0	100	100
All	All	709/709 (100%)	679 (96%)	30 (4%)	33	58

All (30) residues with a non-rotameric sidechain are listed below:

Mol	Chain	Res	Type
1	A	11	ARG
1	A	15	ASN
1	A	30	THR
1	A	74	GLU
1	A	76	ASN
1	A	87	ASN
1	A	179	ILE
1	A	216	ASN
2	B	20	ASN

Continued on next page...

CONFIDENTIAL VALIDATION REPORT

Continued from previous page...

Mol	Chain	Res	Type
2	B	25	THR
2	B	48	THR
2	B	89	MET
2	B	184	ILE
2	B	206	ASN
2	B	239	ILE
2	B	245	VAL
2	B	256	ARG
3	C	40	VAL
3	C	42	ASN
3	C	55	ILE
3	C	59	GLN
3	C	114	LEU
3	C	131	LEU
3	C	149	MET
3	C	152	THR
3	C	168	VAL
3	C	173	GLN
3	C	185	THR
3	C	218	ASN
3	C	238	GLN

Some sidechains can be flipped to improve hydrogen bonding and reduce clashes. All (16) such sidechains are listed below:

Mol	Chain	Res	Type
1	A	57	ASN
1	A	90	GLN
1	A	214	HIS
1	A	216	ASN
1	A	248	GLN
1	A	256	ASN
2	B	20	ASN
2	B	95	ASN
2	B	118	HIS
2	B	167	GLN
2	B	206	ASN
3	C	20	GLN
3	C	105	ASN
3	C	195	GLN
3	C	218	ASN
3	C	233	GLN

CONFIDENTIAL VALIDATION REPORT

5.3.3 RNA (i)

There are no RNA molecules in this entry.

5.4 Non-standard residues in protein, DNA, RNA chains (i)

There are no non-standard protein/DNA/RNA residues in this entry.

5.5 Carbohydrates (i)

There are no carbohydrates in this entry.

5.6 Ligand geometry (i)

2 ligands are modelled in this entry.

In the following table, the Counts columns list the number of bonds (or angles) for which Mogul statistics could be retrieved, the number of bonds (or angles) that are observed in the model and the number of bonds (or angles) that are defined in the Chemical Component Dictionary. The Link column lists molecule types, if any, to which the group is linked. The Z score for a bond length (or angle) is the number of standard deviations the observed value is removed from the expected value. A bond length (or angle) with $|Z| > 2$ is considered an outlier worth inspection. RMSZ is the root-mean-square of all Z scores of the bond lengths (or angles).

Mol	Type	Chain	Res	Link	Bond lengths			Bond angles		
					Counts	RMSZ	$\#\|Z\| > 2$	Counts	RMSZ	$\#\|Z\| > 2$
5	QFW	C	301	-	28,30,30	1.33	2 (7%)	39,43,43	1.25	5 (12%)
6	MYR	D	101	4	14,14,15	0.28	0	13,13,15	1.14	2 (15%)

In the following table, the Chirals column lists the number of chiral outliers, the number of chiral centers analysed, the number of these observed in the model and the number defined in the Chemical Component Dictionary. Similar counts are reported in the Torsion and Rings columns. '-' means no outliers of that kind were identified.

Mol	Type	Chain	Res	Link	Chirals	Torsions	Rings
5	QFW	C	301	-	-	4/15/19/19	0/3/3/3
6	MYR	D	101	4	-	3/11/12/13	-

All (2) bond length outliers are listed below:

Mol	Chain	Res	Type	Atoms	Z	Observed(Å)	Ideal(Å)
5	C	301	QFW	S1-N1	5.26	1.72	1.63

Continued on next page...

CONFIDENTIAL VALIDATION REPORT

Continued from previous page...

Mol	Chain	Res	Type	Atoms	Z	Observed(Å)	Ideal(Å)
5	C	301	QFW	C13-C12	2.12	1.40	1.37

All (7) bond angle outliers are listed below:

Mol	Chain	Res	Type	Atoms	Z	Observed(^{o})	Ideal(^{o})
5	C	301	QFW	C3-C2-C20	-3.60	114.85	120.20
5	C	301	QFW	C8-C7-S1	-2.63	117.83	120.03
6	D	101	MYR	C7-C6-C5	-2.62	101.15	114.42
5	C	301	QFW	C17-O3-C12	-2.38	111.72	117.93
6	D	101	MYR	C12-C11-C10	-2.28	102.84	114.42
5	C	301	QFW	C4-C5-N1	2.26	124.97	120.09
5	C	301	QFW	C7-S1-N1	-2.24	104.01	106.83

There are no chirality outliers.

All (7) torsion outliers are listed below:

Mol	Chain	Res	Type	Atoms
6	D	101	MYR	C1-C2-C3-C4
6	D	101	MYR	C7-C8-C9-C10
6	D	101	MYR	C6-C7-C8-C9
5	C	301	QFW	C16-C7-S1-O2
5	C	301	QFW	C8-C7-S1-O2
5	C	301	QFW	C11-C12-O3-C17
5	C	301	QFW	C13-C12-O3-C17

There are no ring outliers.

1 monomer is involved in 4 short contacts:

Mol	Chain	Res	Type	Clashes	Symm-Clashes
6	D	101	MYR	4	0

The following is a two-dimensional graphical depiction of Mogul quality analysis of bond lengths, bond angles, torsion angles, and ring geometry for all instances of the Ligand of Interest. In addition, ligands with molecular weight > 250 and outliers as shown on the validation Tables will also be included. For torsion angles, if less then 5% of the Mogul distribution of torsion angles is within 10 degrees of the torsion angle in question, then that torsion angle is considered an outlier. Any bond that is central to one or more torsion angles identified as an outlier by Mogul will be highlighted in the graph. For rings, the root-mean-square deviation (RMSD) between the ring in question and similar rings identified by Mogul is calculated over all ring torsion angles. If the average RMSD is greater than 60 degrees and the minimal RMSD between the ring in question and any Mogul-identified rings is also greater than 60 degrees, then that ring is considered an outlier.

CONFIDENTIAL VALIDATION REPORT

The outliers are highlighted in purple. The color gray indicates Mogul did not find sufficient equivalents in the CSD to analyse the geometry.

Ligand QFW C 301

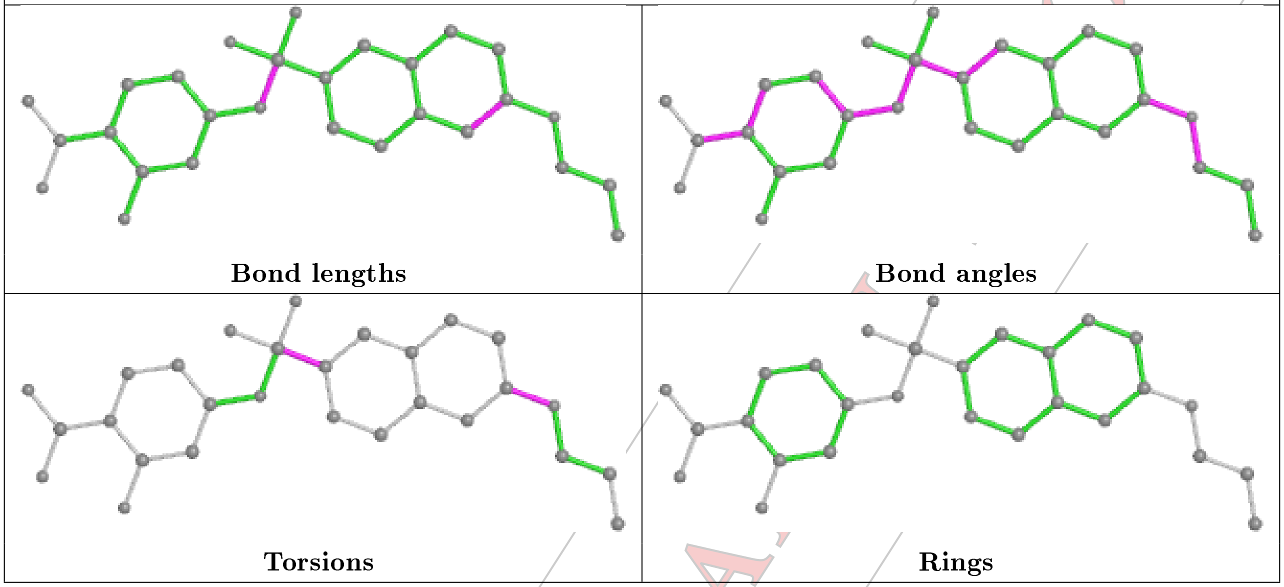

5.7 Other polymers (i)

There are no such residues in this entry.

5.8 Polymer linkage issues (i)

The following chains have linkage breaks:

Mol	Chain	Number of breaks
4	D	2

All chain breaks are listed below:

Model	Chain	Residue-1	Atom-1	Residue-2	Atom-2	Distance (Å)
1	D	13:HIS	C	14:ILE	N	15.51
1	D	11:GLY	C	12:ALA	N	3.36

CONFIDENTIAL VALIDATION REPORT

6 Map visualisation (i)

This section contains visualisations of the EMDB entry EMD-11165. These are intended to permit visual inspection of the internal detail of the map and identification of artifacts.

6.1 Orthogonal projections (i)

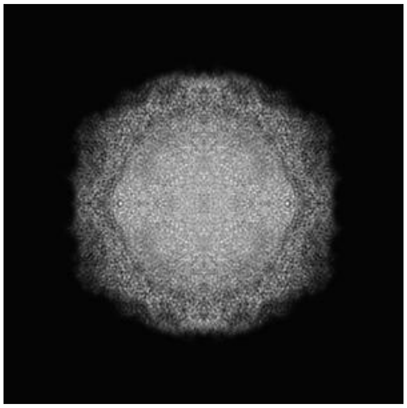
X

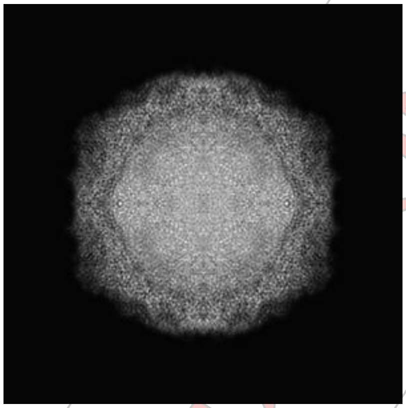
Y

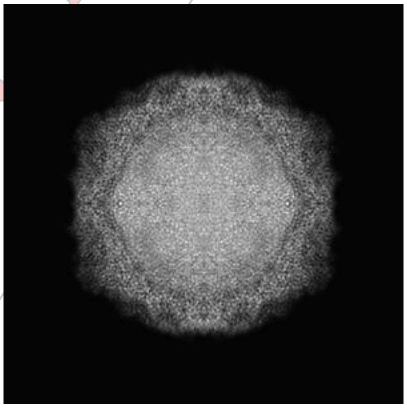
Z

The images above show the map projected in three orthogonal projections, in greyscale.

6.2 Central slices (i)

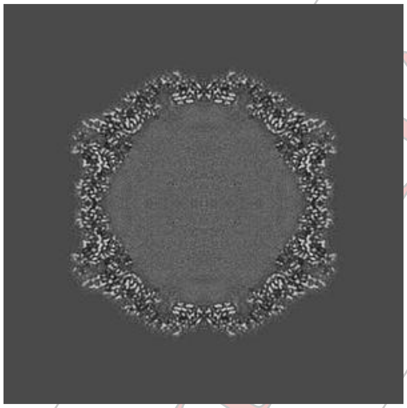
X

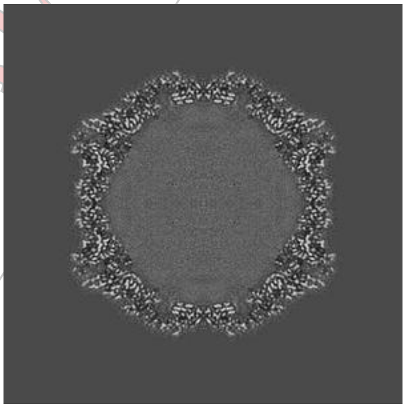
Y

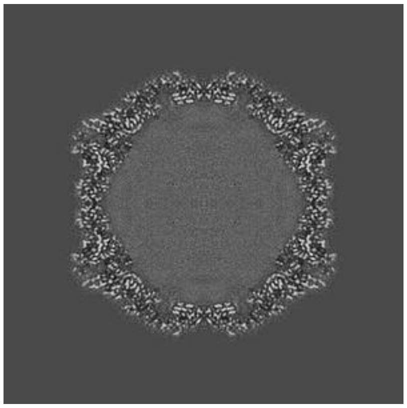
Z

The images above show central slices of the map in three orthogonal directions, in greyscale.

CONFIDENTIAL VALIDATION REPORT

6.3 Largest variance slices (i)

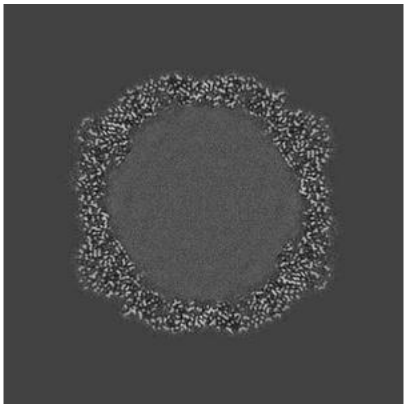

X Index: 203

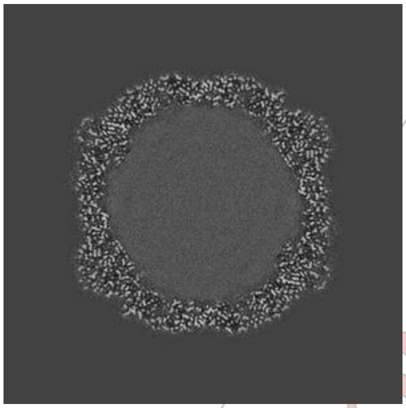

Y Index: 203

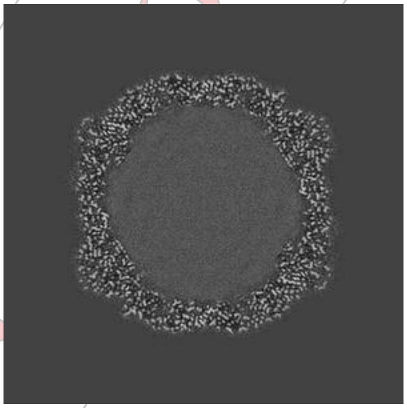

Z Index: 203

The images above show the highest variance slices of the map in three orthogonal directions, in greyscale.

6.4 Orthogonal surface views (i)

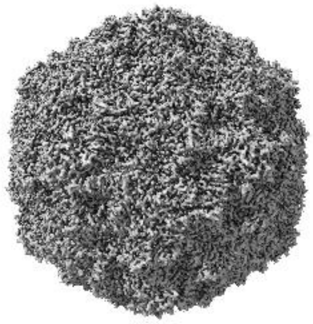

X

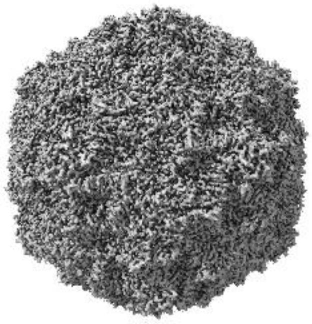

Y

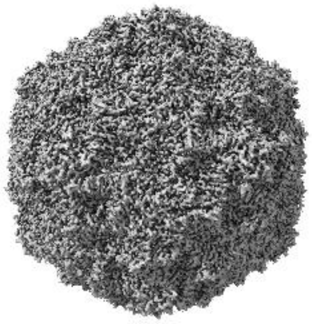

Z

The images above show the 3D surface view of the map at the recommended contour level 0.00928. This in conjunction with the slice images can indicate whether an appropriate contour level has been selected.

6.5 Mask visualisation (i)

This section was not generated. No masks were provided.

CONFIDENTIAL - VALIDATION REPORT

7 Map analysis (i)

This section contains the results of statistical analysis of the map.

7.1 Map-value distribution (i)

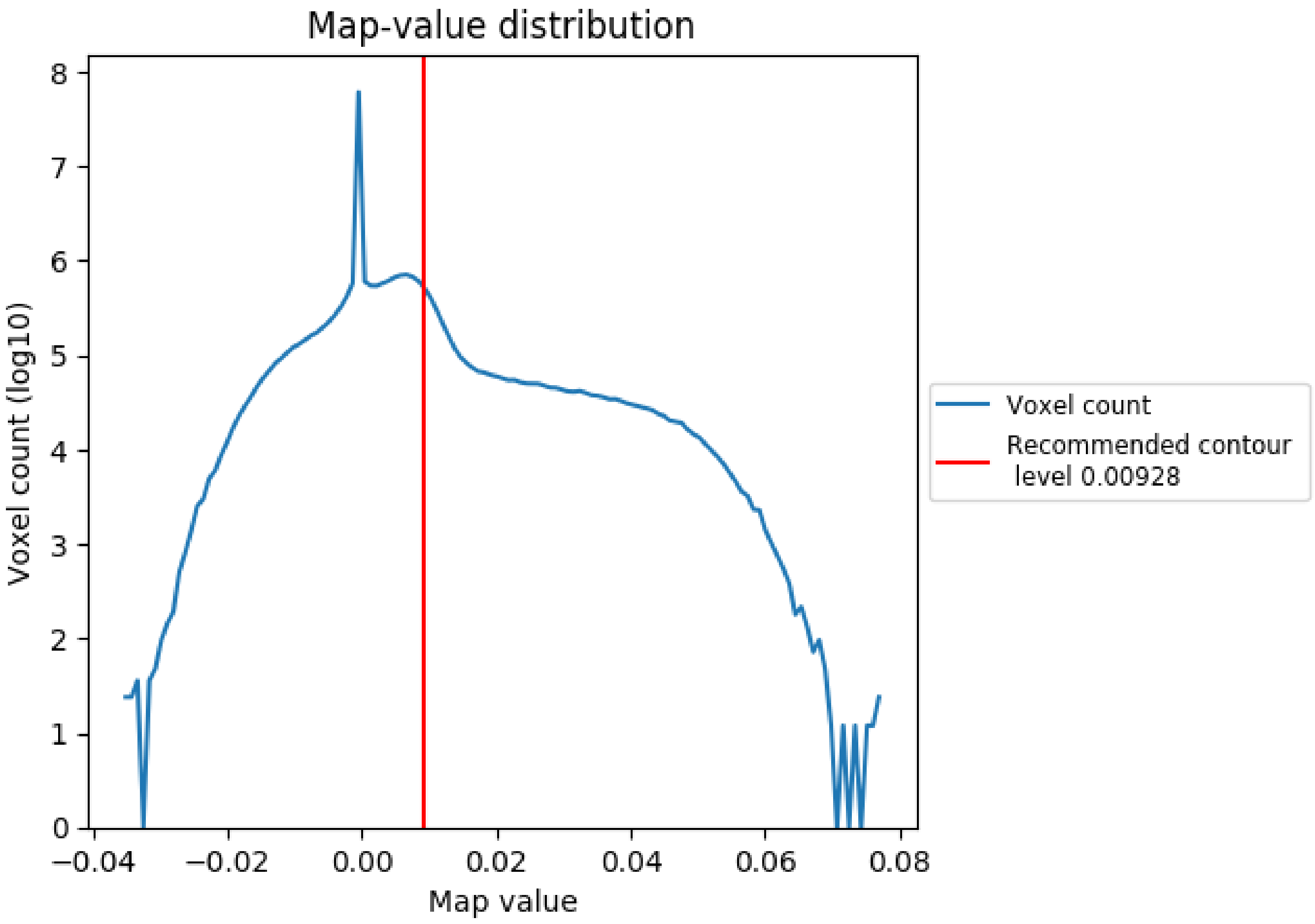

The map-value distribution is plotted in 128 intervals along the x-axis. The y-axis is logarithmic. A spike in this graph at zero usually indicates that the volume has been masked.

CONFIDENTIAL REPORT

7.2 Volume estimate ⓘ

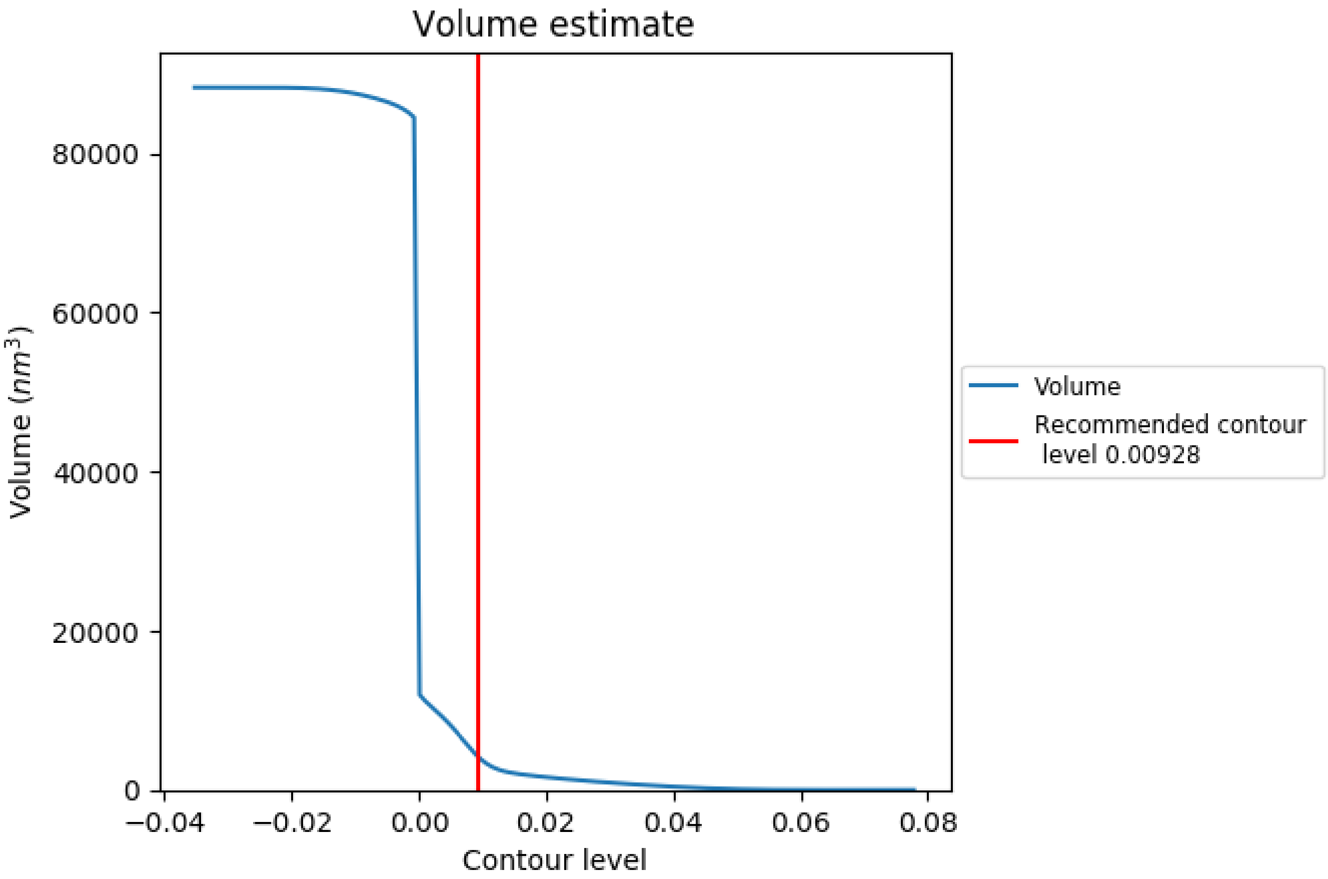

The volume at the recommended contour level is 4235 nm^3; this corresponds to an approximate mass of 3826 kDa.

The volume estimate graph shows how the enclosed volume varies with the contour level. The recommended contour level is shown as a vertical line and the intersection between the line and the curve gives the volume of the enclosed surface at the given level.

CONFIDENTIAL REPORT

7.3 Rotationally averaged power spectrum ⓘ

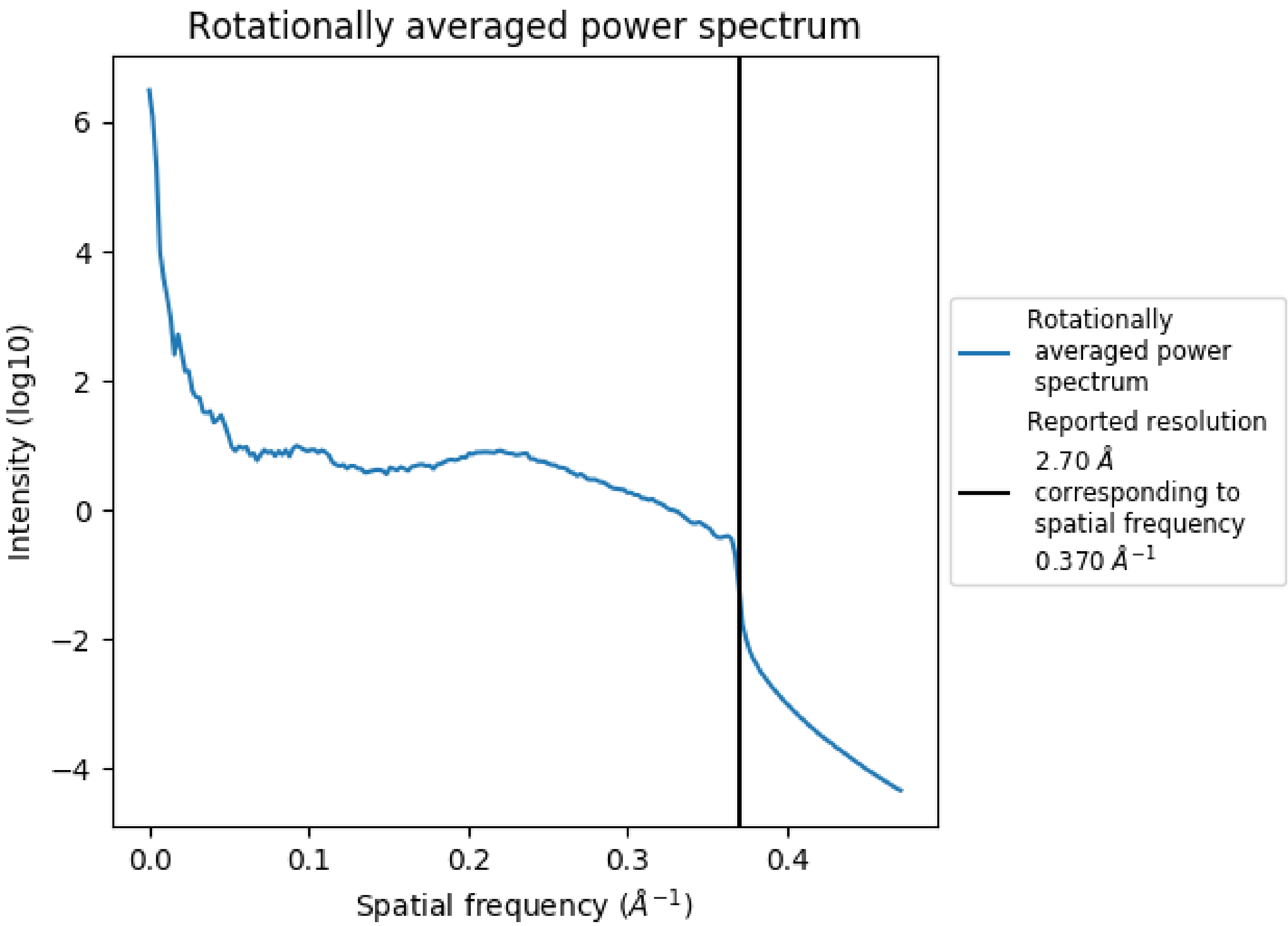

CONFIDENTIAL REPORT

8 Fourier-Shell correlation (i)

This section was not generated. No FSC curve or half maps provided.

CONFIDENTIAL VALIDATION REPORT

9 Map-model fit (i)

This section contains information regarding the fit between EMDB map EMD-11165 and PDB model 6ZCK. Per-residue inclusion information can be found in section 3 on page 6.

9.1 Map-model overlay (i)

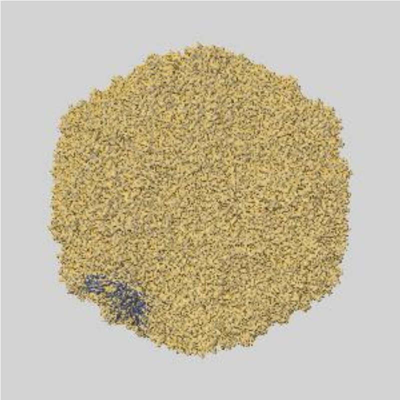
X

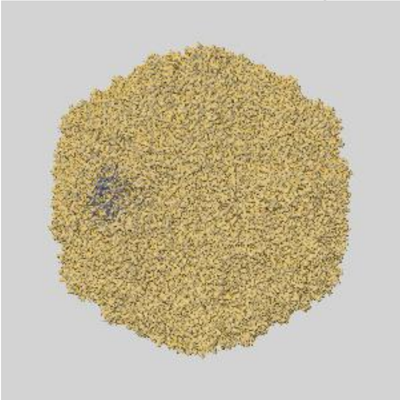
Y

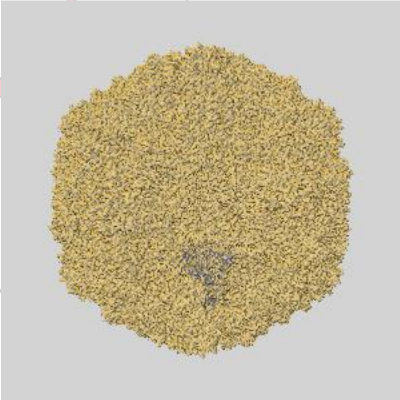
Z

The images above show the 3D surface view of the map at the recommended contour level 0.00928 at 50% transparency in yellow overlaid with a ribbon representation of the model coloured in blue. These images allow for the visual assessment of the quality of fit between the atomic model and the map.

CONFIDENTIAL VALIDATION REPORT

9.2 Atom inclusion (i)

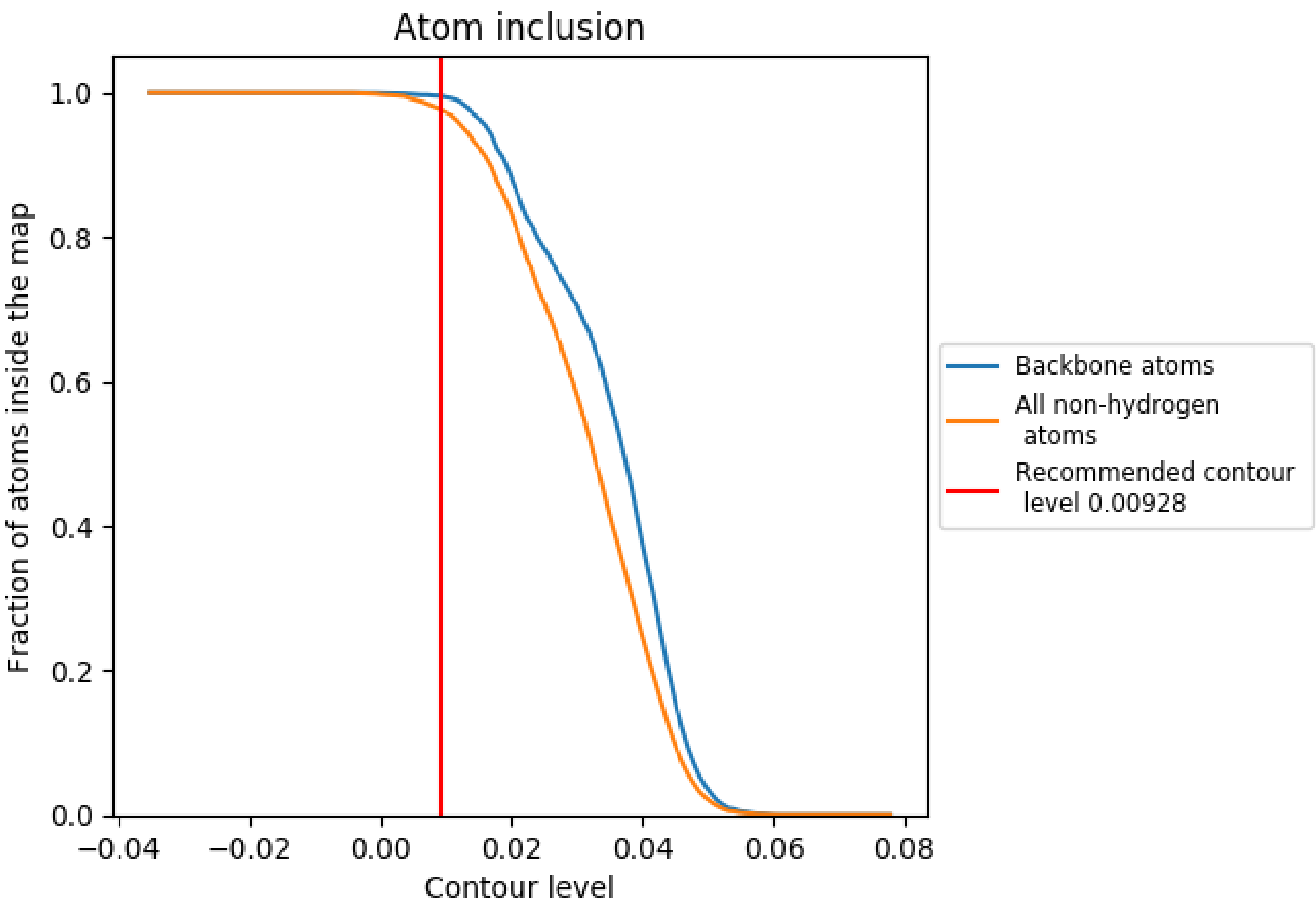

At the recommended contour level, 100% of all backbone atoms, 98% of all non-hydrogen atoms, are inside the map.

CONFIDENTIAL
RT
