# Supplementary material for: Identification of a conserved virion-stabilizing network inside the interprotomer pocket of enteroviruses
Source: Commun Biol. 2021 Feb 26;4:250. doi: 10.1038/s42003-021-01779-x (PMC7910612; doi:10.1038/s42003-021-01779-x)
Supplement: Supplementary file 3 — Description of Additional Supplementary Files [file 42003_2021_1779_MOESM3_ESM.pdf]

Description of Additional Supplementary Files

File Name: Supplementary Data 1
Description: Table of CVB4 infectivity in the presence or absence of CP48 after exposure to increasing temperature.
